# Human milk-associated bacterial communities associate with the infant gut microbiome over the first year of life

**DOI:** 10.3389/fmicb.2023.1164553

**Published:** 2023-04-17

**Authors:** Sara N. Lundgren, Juliette C. Madan, Margaret R. Karagas, Hilary G. Morrison, Brock C. Christensen, Anne G. Hoen

**Affiliations:** ^1^Department of Epidemiology, Geisel School of Medicine at Dartmouth, Lebanon, NH, United States; ^2^Department of Pediatrics, Children’s Hospital at Dartmouth, Lebanon, NH, United States; ^3^Department of Psychiatry, Children’s Hospital at Dartmouth, Lebanon, NH, United States; ^4^Department of Community and Family Medicine, Geisel School of Medicine at Dartmouth, Lebanon, NH, United States; ^5^Josephine Bay Paul Center, Marine Biological Laboratory, Woods Hole, MA, United States; ^6^Department of Molecular and Systems Biology, Geisel School of Medicine at Dartmouth, Hanover, NH, United States; ^7^Department of Biomedical Data Science, Geisel School of Medicine at Dartmouth, Lebanon, NH, United States; ^8^Department of Microbiology and Immunology, Geisel School of Medicine at Dartmouth, Lebanon, NH, United States

**Keywords:** infant gut microbiome, breast milk microbiome, cesarean delivery, breastfeeding, microbial co-occurrence

## Abstract

**Introduction:**

Microbial communities inhabiting the human infant gut are important for immune system development and lifelong health. One critical exposure affecting the bacterial colonization of the infant gut is consumption of human milk, which contains diverse microbial communities and prebiotics. We hypothesized that human milk-associated microbial profiles are associated with those of the infant gut.

**Methods:**

Maternal–infant dyads enrolled in the New Hampshire Birth Cohort Study (*n* = 189 dyads) contributed breast milk and infant stool samples collected approximately at 6 weeks, 4 months, 6 months, 9 months, and 12 months postpartum (*n* = 572 samples). Microbial DNA was extracted from milk and stool and the V4-V5 region of the bacterial 16S rRNA gene was sequenced.

**Results:**

Clustering analysis identified three breast milk microbiome types (BMTs), characterized by differences in *Streptococcus*, *Staphylococcus*, *Pseudomonas*, *Acinetobacter*, and microbial diversity. Four 6-week infant gut microbiome types (6wIGMTs) were identified, differing in abundances of *Bifidobacterium*, *Bacteroides*, *Clostridium*, *Streptococcus*, and *Escherichia*/*Shigella*, while two 12-month IGMTs (12mIGMTs) differed primarily by *Bacteroides* presence. At 6 weeks, BMT was associated with 6wIGMT (Fisher’s exact test value of *p* = 0.039); this association was strongest among infants delivered by Cesarean section (Fisher’s exact test value of *p* = 0.0028). The strongest correlations between overall breast milk and infant stool microbial community structures were observed when comparing breast milk samples to infant stool samples collected at a subsequent time point, e.g., the 6-week breast milk microbiome associated with the 6-month infant gut microbiome (Mantel test *Z*-statistic = 0.53, value of *p* = 0.001). *Streptoccous* and *Veillonella* species abundance were correlated in 6-week milk and infant stool, and 4- and 6-month milk *Pantoea* species were associated with infant stool *Lachnospiraceae* genera at 9 and 12  months.

**Discussion:**

We identified clusters of human milk and infant stool microbial communities that were associated in maternal–infant dyads at 6 weeks of life and found that milk microbial communities were more strongly associated with infant gut microbial communities in infants delivered operatively and after a lag period. These results suggest that milk microbial communities have a long-term effect on the infant gut microbiome both through sharing of microbes and other molecular mechanisms.

## 1. Introduction

Breastfeeding is an important exposure influencing the colonization patterns of bacteria in the infant intestinal tract (Azad et al., 2013; Madan et al., 2016). Human milk is a complex mixture including numerous biologically active components including immune cells (Field, 2005), fucosylated and sialylated oligosaccharides (Grönberg et al., 1992; Thurl et al., 2010), fatty acids (Koletzko et al., 1988), micro-RNAs (Kosaka et al., 2010; Zhou et al., 2012), and live microbes (Jost et al., 2013). These microbial communities are highly variable between individuals and are thought to be influenced by factors such as maternal body mass, delivery mode, pumping of breast milk, antibiotic exposure, and maternal diet (Cabrera-Rubio et al., 2012; Khodayar-Pardo et al., 2014; Hermansson et al., 2019; Moossavi et al., 2019; Padilha et al., 2019; LeMay-Nedjelski et al., 2021), as well as by infant factors such as pacifier usage (Sindi et al., 2023) and whether a baby has ever latched (Kordy et al., 2020). Current evidence suggests that these bacteria along with those colonizing the skin around the mother’s nipple may be transferred to and may colonize the infant gut through breastfeeding (Solis et al., 2010; Pannaraj et al., 2017; Biagi et al., 2018; Bogaert et al., 2023), including evidence from preterm infants showing associations between the overall microbial profile of human milk and both the infant gut and oral microbiomes (Biagi et al., 2018). The vertical transfer of bacteria in breast milk to the infant gut appears to be facilitated by secretory immunoglobulin A coating of milk bacteria (Qi et al., 2022). However, observational studies cannot rule out the possibility that shared microbes originate from a shared exposure for the mother and infant. Understanding how the microbial communities in breast milk affect those in the infant gut is critical given that the early life infant gut microbiome plays a key role in infant development and long-term health, including production of immunoglobulins and cytokines (Sjogren et al., 2009), development of immune-mediated conditions such as atopic eczema (Abrahamsson et al., 2012), as well as body mass index (BMI) in childhood (Stanislawski et al., 2018).

Most studies investigating the association of breast milk microbiota with infant gut microbiota have focused on assessing the co-occurrence of microbes in breast milk and the infant gut in maternal–infant dyads in contemporaneously collected samples. In order to capture an infant’s full microbial exposure during breastfeeding, we did not use an aseptic collection process for milk samples; we refer to the microbiome measured in milk samples as the breast milk microbiome, although it likely also contains maternal skin-associated microbes. We analyzed the human milk and infant gut microbiomes in 189 mother-infant dyads from the New Hampshire Birth Cohort Study (NHBCS) over the first year of life using targeted sequencing of the 16S rRNA gene to assess the complex relationships between the microbial communities found in human milk and the infant gut over time.

## 2. Materials and methods

### 2.1. Study population

All subjects were participants in the NHBCS, a large epidemiologic study which has been defined in previous work (Gilbert-Diamond et al., 2011; Farzan et al., 2013). Briefly, pregnant women between the ages of 18 and 45 years who lived at a home that relied on water from a private water system and are received prenatal care in clinics in New Hampshire, United States, were recruited at routine prenatal appointments. Eligible subjects were enrolled between 24 and 28 weeks of gestation. All study methods were executed in agreement with guidelines approved by the Center for the Protection of Human Subjects at Dartmouth, the institutional review board. Written informed consent for participation of themselves and their infants was obtained from all subjects.

Every 4 months, subjects completed telephone interviews on breastfeeding and formula supplementation, infant infections and exposure to antibiotics, maternal medications, and other information. Infants who were currently breastfed and had never received formula at the time of stool collection were classified as unexposed to formula, while infants who had ever received formula before stool collection were classified as exposed to formula. Upon study entry, subjects completed a self-administered questionnaire including information on height, pre-pregnancy weight, race, education, and parity. If self-reported measures of height and weight were unavailable, measures from prenatal medical records were used to estimate pre-pregnancy BMI. Prenatal medical records were also used to ascertain gestational age, which was estimated from date of last menstrual period and ultrasound, and prenatal antibiotics exposure. Weight gain during pregnancy was self-reported by subjects in the postpartum questionnaire. Maternal delivery medical records were reviewed to determine delivery mode and peripartum antibiotic exposure, while infant perinatal antibiotic exposure was determined from the infant medical record.

### 2.2. Sample collection and DNA extraction

Breast milk and infant stool samples were collected at home by study participants and brought on cold packs to the postpartum follow-up appointment or to regularly scheduled infant well child visits at approximately 6 weeks, 4 months, 6 months, 9 months, and 12 months of age. The median (minimum, 1^st^ quartile, 3^rd^ quartile, maximum) number of days from the target collection time was 3 days (min = −16 days, 1^st^ quartile = 0 days, 3^rd^ quartile = 6 days, max = 79 days) for 6-week infant stool, 7 days (min = −11 days, 1^st^ quartile = 2 days, 3^rd^ quartile = 12 days, max = 60 days) for 4-month infant stool, 6 days (min = −57 days, 1^st^ quartile = 3 days, 3^rd^ quartile = 24 days, max = 45 days) for 6-month infant stool, 9.5 days (min = −8 days, 1^st^ quartile = 3 days, 3^rd^ quartile = 16.8 days, max = 35 days) for 9-month infant stool, 7 days (min = −32 days, 1^st^ quartile = −3 days, 3^rd^ quartile = 24 days, max = 209 days) for 12-month infant stool, 3 days (min = −23 days, 1^st^ quartile = 0 days, 3^rd^ quartile = 6 days, max = 122 days) for 6-week breast milk, 6 days (min = −23 days, 1^st^ quartile = 2.3 days, 3^rd^ quartile = 10.8 days, max = 60 days) for 4-month breast milk, 10.5 days (min = −28 days, 1^st^ quartile = 2.3 days, 3^rd^ quartile = 25.5 days, max = 44 days) for 6-month breast milk, 9 days (min = −8 days, 1^st^ quartile = 3 days, 3^rd^ quartile = 15.5 days, max = 35 days) for 9-month breast milk, and 9 days (min = 0 days, 1^st^ quartile = 5.5 days, 3^rd^ quartile = 13.5 days, max = 34 days) for 12-month breast milk, with the majority of samples being collected close to the target time point (Supplementary Figure S1).

Milk was collected from bilateral breasts *via* either manual expression or pumping, with separate collection bottles used for milk from each breast, and study-provided diapers were used to collect infant stool. Breast tissue was not sterilized prior to milk sample collection in order to accurately capture an infant’s microbial exposure from breast milk, either *via* breastfeeding or bottle-feeding of expressed breast milk. Subjects provided between 18 and 80 ml of milk per breast, with a median of 35 ml. The majority of samples were collected during the day before noon, with a median collection time of 9: 20 AM (1^st^ quartile = 7:30 AM, 3^rd^ quartile = 12:15 PM, Supplementary Figure S2) and within 1 h after the last feed (Supplementary Figure S3). Samples were immediately chilled upon acquisition. Within 24 h of receipt, milk and stool samples were processed. Briefly, 3 ml of whole milk from each breast were mixed and centrifuged for 90 min at 5000 rpm. The temperature was maintained at 4°C. After removing the fat and supernatant, 150 μl of PBS was added to the cell pellet and thoroughly mixed before transferring to a 1.5 ml Eppendorf tube and freezing at−20°C. As previously described (Lundgren et al., 2018), stool samples were aliquoted and stored frozen at− 80°C. Samples were thawed at room temperature prior to microbial DNA extraction using the Zymo DNA extraction kit (Zymo Research) and established methods (Wu et al., 2010). DNA was quantified using OD260/280 Nanodrop.

### 2.3. Targeted 16S rRNA sequencing

The Marine Biological Laboratory (MBL) in Woods Hole, MA performed microbial DNA sequencing. We sequenced the V4-V5 hypervariable region of the bacterial 16S rRNA gene, which was chosen in part for its ability to capture bacteria that have a known important role in the infant gut microbiome such as *Bifidobacterium*, and its lower intra-sample variability compared to other commonly sequenced hypervariable regions (Kozich et al., 2013). Using established methods (Huse et al., 2014; Newton et al., 2015), 16S rDNA V4-V5 amplicons were generated from purified genomic DNA samples using fusion primers (Forward Primer: (518F) CCAGCAGCYGCGGTAAN; Reverse Primers: (926R) CCGTCAATTCNTTTRAGT, CCGTCAATTTC TTTGAGT, CCGTCTATTCCTTTGANT). Forward primers containing one of eight five-nucleotide barcodes between the Illumina-specific bridge and sequencing primer regions and the 16S-specific region and a single reverse primer containing one of 12 Illumina indices allowed for multiplexing of 96 samples per lane. Amplifications were done in triplicate with one negative control for internal quality control at the MBL. Additionally, positive and extraction controls were prepared with samples at Dartmouth for sequencing. qPCR (Kapa Biosystems) was used to quantify the amplicon pool. Each pool of 96 libraries was sequenced using One Illumina MiSeq 500 cycle paired end run. The microbial sequencing data are available through NCBI GenBank under the accession number PRJNA296814. There were no sequence reads detected from extraction controls.

### 2.4. Data processing

Primers were removed from forward and reverse reads using the cutadapt program (Martin, 2011). All subsequent sequence processing and statistical analyzes were completed in R version 3.5.0 (R Core Team, 2016). We used the R package, DADA2 (version 1.4), to identify amplicon sequence variants (ASVs) from sequencing reads, which uses a Bayesian method to correct for amplicon sequencing errors and identifies and removes chimeric reads (Callahan et al., 2016). We constructed a phylogenetic tree using the R package phangorn (Schliep, 2010).

The median (minimum, 1^st^ quartile, 3^rd^ quartile, maximum) read depth observed was 66,587 (min = 1,115, 1^st^ quartile = 24,019, 3^rd^ quartile = 110,543, max = 1,429,834) for breast milk and 97,799 (min = 15,900, 1^st^ quartile = 79,482, 3^rd^ quartile = 127,636, max = 676,957) for infant stool. As a sensitivity analysis we compared results rarefying to the minimum sequencing depth, 1,115 for breast milk and 15,900 for infant stool. Compared with un-rarefied data, we found no significant difference in results of alpha diversity for rarefied data; therefore, read counts were not rarefied in the main analyzes to prevent discarding important microbial sequencing information.

### 2.5. Selection of subjects

We included maternal–infant dyads with at least one milk and one stool sample collected, where the stool sample was collected at the same or a subsequent timepoint (i.e., stool sample not collected before milk collection). We removed samples with fewer than 1,000 reads (*n* = 4), and outliers based on alpha diversity and beta diversity (*n* = 8). This resulted in 189 maternal–infant dyads, totaling 572 milk and infant stool samples (Supplementary Table S1).

### 2.6. Statistical analysis

#### 2.6.1. Comparison of microbiome composition in breast milk and the infant gut

In order to compare the microbial composition of human milk and infant stool over the first year of life, we calculated relative abundances of ASVs and summed abundances of ASVs with a common taxonomic assignment together. A heatmap of the relative abundance of the top 25 most abundant microbial taxa on average was made using the R package *pheatmap*.

#### 2.6.2. Alpha diversity analysis

Next, we assessed the alpha diversity of infant gut and human milk microbial communities using three common alpha diversity metrics: Simpson’s diversity index (SDI), Shannon diversity index (ShDI), and the observed number of unique ASVs detected in a sample (observed ASVs). SDI was transformed using the logit transformation due to having a skewed distribution. Differences in alpha diversity in milk (*n* = 245) and the infant gut (*n* = 327) across the first year of life were tested using linear models with a random effect for subject; value of *p*s were approximated using the Kenward-Roger method. Results from analyzes using estimates of alpha diversity after rarefaction of the data did not differ and are included as supplemental data.

#### 2.6.3. Proportion of infant gut reads overlapping with milk ASVs

Next, we calculated the fraction of infant gut microbiome reads from ASVs that were also present in milk collected from the infant’s mother at the same time as or before collection of the infant stool. We assessed this ASV overlap in all dyads and in the subset of dyads where the infant was unexposed to formula at the time of stool collection, as we hypothesized that a reduced exposure to breast milk due to supplementation with formula could affect the extent of overlap between breast milk and infant stool microbiota. We also repeated this analysis stratified by delivery mode and infant sex as we have previously found these to be important effect modifiers of exposures of interest on the infant gut microbiome. We compared the distribution of observed ASV overlap with the ASV overlap occurring between random mother-infant dyads at the same timepoint using the Kolmogorov–Smirnov test. Including every mother-infant dyad, including multiple observations of the same dyad at differing timepoint pairs when available (for example, overlap between 6-week breast milk and 6-week infant stool as well as between 6-week breast milk and 4-month infant stool), we modeled the associations of exposure to formula, delivery mode, and infant sex with the proportion of reads in the infant gut from ASVs in breast milk using linear mixed effects models to account for repeated measures across the first year of life; value of *p*s were approximated using the Kenward-Roger method. We performed both unadjusted models, in addition to a fully adjusted model including all examined variables as well as the age of the infant at the time of stool collection. The maximum number of dyad timepoint-pairs was 437, however some data on formula exposure was missing, resulting in 397 dyad timepoint-pairs for analyzes including assessment of formula exposure.

#### 2.6.4. Clustering analysis

The unsupervised clustering method partitioning around medoids (PAM) was used to identify patterns of infant gut microbiota (infant gut microbiome types, IGMTs) and human milk microbiota (breast milk microbiome types, BMTs), respectively using the generalized UniFrac distance (GUniFrac), a phylogenetically-based distance metric that moderately weights both rare and abundant ASVs, and displays increased statistical power with unrarefied data (Chen et al., 2012; Lundgren et al., 2019). We chose this method of clustering since we have previously used it to identify biologically meaningful patterns of infant gut microbiota and human milk microbiota in the NHBCS (Lundgren et al., 2018, 2019). We assessed the quality of clustering using the gap statistic as well as the observed separation on principal coordinates plots. The gap statistic tended to indicate a very high number of clusters for infant gut samples, so we chose the lowest number of clusters necessary to have the least overlap on principal coordinates analysis (PCoA) plots.

Clustering was performed within sample types only. The clustering of infant stool samples was performed three ways, (1) including samples from all timepoints to identify infant gut microbiome type (*n* = 327, IGMT), (2) including only 6-week stool samples to identify 6-week infant gut microbiome type (*n* = 151, 6wIGMT), and (3) including only 12-month stool samples to identify 12-month infant gut microbiome type (*n* = 113, 12mIGMT). The reason for restricting the clustering to 6-week and 12-month timepoints was to prevent clusters that were primarily comprised of 6-week to 9-month samples or 12-month samples, respectively, from containing only one two samples in subsequent analyzes focusing on the 6-week or 12-month timepoints separately. Breast milk samples were clustered two ways, (1) including all breast milk samples to identify breast milk microbiome type (*n* = 245, BMT) and (2) including only 6-week breast milk samples to identify 6-week breast milk microbiome type (*n* = 181, 6wBMT). The clustering including only 6-week samples was used for the analyzes including only 6-week breast milk samples testing the association between infant gut cluster and breast milk cluster since the infant gut microbiome clusters used were also clustered using only 6-week infant gut samples, while the clustering including all breast milk samples was used to assess the stability of breast milk clusters over time. We compared Simpson’s diversity index between clusters using the Kruskal-Wallis rank-sum test. Additionally, we classified samples based on their most abundant taxon.

#### 2.6.5. Stability of the breast milk and infant stool microbiomes over time

There were 25 subjects from which we collected longitudinal breast milk samples, ranging from two to five samples over the first year postpartum (*n* = 81 samples). In order to assess the stability of breast milk microbiota over time, which may have implications for the effect on the infant gut microbiome and development, we examined whether subjects who provided longitudinal samples always belonged to the same BMT, or if the BMT changed over time. Each subject was classified into one of three categories, to reflect higher or lower stability of microbial populations over time: (0) all of the samples provided by the subject belong to the same cluster, (1) samples provided by a single subject were classified to two different clusters, or (2) a subjects’ samples were classified to three clusters at different time points.

The same criteria were used to categorize infant stool samples for the 27 subjects with at least two longitudinally collected samples through the first 9 months of life (*n* = 81 samples, excluding 12-month samples), except that 3 included subjected classified to 3 *or more* clusters through the first 9 months of life. We excluded the 12-month infant stool sampling timepoint since 12-month stool samples clustered separately from 6-week through 9-month samples, reflecting normal changes in the infant gut microbiome over the first year of life.

In order to identify maternal and infant factors associated with the stability of breast milk and infant gut clusters and of the most abundant taxon over the first year, we used the Kruskal-Wallis rank-sum test for continuous variables and Fisher’s exact test for categorical variables. We tested the relation between breast milk cluster-switching and maternal age, pre-pregnancy BMI and gestational weight gain, parity, prenatal, peripartum, and postpartum antibiotics, infant sex, and the number days postpartum the last sample was collected, and tested the relation between infant stool microbiome type switching and maternal pre-pregnancy BMI and gestational weight gain, parity, maternal prenatal or peripartum antibiotics, infant antibiotics at delivery, delivery mode, infant sex, and the age of the baby when the last stool sample was collected. Since the timing of sample collection for the last sample may confound the association between variables of interest and cluster switching, we performed multinomial logistic regression with the degree of cluster switching as the outcome (no cluster switching as the reference category) and maternal and infant variables as predictors, adjusting for the timing of the last collected sample. Variables which showed some evidence of association with cluster switching (value of *p* < 0.1) were retained in the fully adjusted models. To visualize the relationship between cluster switching and variables of interest, we plotted the predicted probability of cluster switching degree by variables associated with cluster switching degree.

#### 2.6.6. Association between the breast milk and infant gut microbiome clusters

Associations between 6wBMT versus 6wIGMT (*n* = 144 dyads) or 12mIGMT (*n* = 108 dyads) in maternal–infant dyads were tested using the Fisher’s exact test. Cluster associations were tested for all dyads, restricted to infants unexposed to formula at the time of stool collection, as well as stratified by delivery mode and by infant sex. In the same manner, we tested the relation between the most abundant taxon in 6-week breast milk and 6-week and 12-month infant stool in maternal–infant dyads. We were not able to test these associations for intermediate timepoints, such as between 6wBMT and 4-month IGMT due to sample size constraints.

#### 2.6.7. Correlation of overall breast milk microbiome profile with infant gut microbiome profile

To assess the association between the overall microbial community structure of human milk with that of the infant gut, we used the mantel test with 10,000 permutations using GUniFrac distance matrices. This test computes the correlation between two distance matrices, in this case one reflecting breast milk microbial similarity and one reflecting infant stool microbial similarity. We considered that breastfeeding exclusivity, delivery mode, maternal pre-pregnancy BMI, maternal age, exposure to antibiotics during pregnancy, and infant sex could confound the association between breast milk microbiota and infant gut microbiota. In order to control for possible confounding by these factors, we adjusted the analysis by a third distance matrix computed from these variables; categorical variables were coded to be in binary format, and the pairwise Euclidean distance was calculated. We tested both cross-sectional and longitudinal associations, such as between 6-week milk and 6-week stool, and between 6-week milk and 6-month stool. This also included intermediate breast milk time points of 4 months, 6 months, and 9 months. To assess effect modification by known and hypothesized factors, we restricted to infants unexposed to formula at the time of stool collection, to vaginally delivered infants, and by infant sex. We only tested the association if there were at least 10 maternal–infant dyads.

#### 2.6.8. Taxa-taxa correlations

Finally, we tested correlations between the relative abundance of microbial taxa in breast milk and in infant stool. We calculated the relative abundance of each ASV in each sample and filtered the data to only include ASVs where more than 10% of subjects have a relative abundance of more than 0.5% for each sample type and timepoint. Relative abundances were mean centered and scaled by the standard deviation. We computed the Spearman correlation between each breast milk and infant stool taxon. We considered a *q*-value <0.1 to be statistically significant (Benjamini and Hochberg, 1995). To assess an overall measure of correlation for milk and infant stool taxa between different timepoints, we calculated the average magnitude of the Spearman correlation for each timepoint pair.

## 3. Results

### 3.1. Subject characteristics

Maternal and infant characteristics for the 189 mother-infant dyads are reported in Table 1. The average maternal age at study enrollment was 31.9 years; over 96% of participants were non-Hispanic white. 56% of subjects had a normal pre-pregnancy BMI, while over 40% were considered overweight or obese. Just under half of subjects were first-time mothers, and 73% of deliveries were vaginal. Nearly 20% of mothers took antibiotics during pregnancy, and approximately 50% received peripartum antibiotics. In the postpartum period, maternal antibiotic exposure was 10, 15, and 20% at 4, 8, and 12 months after delivery. Slightly over half of the infants in our study were male (52.9%), with an average gestational age of 39.3 weeks and average birth weight of 3,470 grams. Only 2% of infants received antibiotics immediately after delivery, and 3, 14, and 30% were exposed to systemic antibiotics by 4, 8, and 12 months of life, respectively. 60% of infants in this study were unexposed to formula up to 4 months of age. At the time of 6-week stool sample collection, 73% of infants were known to be unexposed to formula, while 11% had received both breast milk and formula and 14% were unknown (Supplementary Table S2). By the 4-month sample collection, 26% of infants had begun receiving solid food in addition to breast milk and formula, and by the 6-month sample collection 80% of infants had begun eating solid food while only 10% of infants were unexposed to formula and 5% had received only breast milk and formula. By the 12-month stool sample collection, 86% of infants were known to have begun eating solid food and the remaining 14% had this data missing.

**Table 1:** Subject characteristics (*n* = 189 mother-infant dyads).

Maternal variables	*N* (%) or Mean [Range]
Maternal age	31.9 [20–43.7]
Pre-pregnancy BMI^a^	25.8 [17.4–44.2]
Underweight	4 (2.1)
Normal	105 (55.6)
Overweight	44 (23.3)
Obese	35 (18.5)
Gestational weight gain (lbs)^b^	31.9 [−4–75]
Parity^a^	1.8 [1–5]
One	90 (47.6)
Two or more	98 (51.9)
Delivery mode	
Vaginal	138 (73.0)
Cesarean section	51 (27.0)
Race	
Non-Hispanic White	181 (95.8)
Asian	1 (0.5)
Black	1 (0.5)
Hispanic White	6 (3.2)
Education^c^	
High school graduate or equivalent	16 (8.5)
Some college or technical school	28 (14.8)
College graduate	64 (33.9)
Any post-graduate schooling	74 (39.2)
Prenatal antibiotics^d^	
No	135 (71.4)
Yes	36 (19.0)
Peripartum antibiotics^c^	
No	88 (46.6)
Yes	94 (49.7)
Antibiotics by 4 months postpartum^e^	
No	140 (74.1)
Yes	20 (10.6)
Antibiotics by 8 months postpartum^f^	
No	119 (63)
Yes	30 (15.9)
Antibiotics by 8 months postpartum^g^	
No	102 (54)
Yes	41 (21.7)
Infant variables	*N* (%) or Mean [Range]
Sex	
Female	89 (47.1)
Male	100 (52.9)
Perinatal antibiotics^h^	
No	176 (93.1)
Yes	4 (2.1)
Gestational age at birth (weeks)	39.3 [30–43.4]
Birth weight (grams)^h^	3466.5 [2296–4,710]
Antibiotics by 4 months of age^i^	
No	155 (82)
Yes	6 (3.2)
Antibiotics by 8 months of age^j^	
No	123 (65.1)
Yes	27 (14.3)
Antibiotics by 12 month of age^g^	
No	87 (46)
Yes	56 (29.6)
Feeding method through 4 months of age^k^	
Unexposed to formula	113 (59.8)
Exposed to formula	51 (27.0)
Feeding method through 8 months of age^l^	
Unexposed to formula	79 (41.8)
Exposed to formula	77 (40.7)
Feeding method through 12 months of age^f^	
Unexposed to formula	42 (22.2)
Exposed to formula	107 (56.6)

Number of subjects with missing data: ^a^1, ^b^2, ^c^7, ^d^18, ^e^29, ^f^40, ^g^46, ^h^9, ^i^28, ^j^39, ^k^25, ^l^33.

### 3.2. Taxonomic composition of breast milk and infant gut microbiota

**Figure 1:**
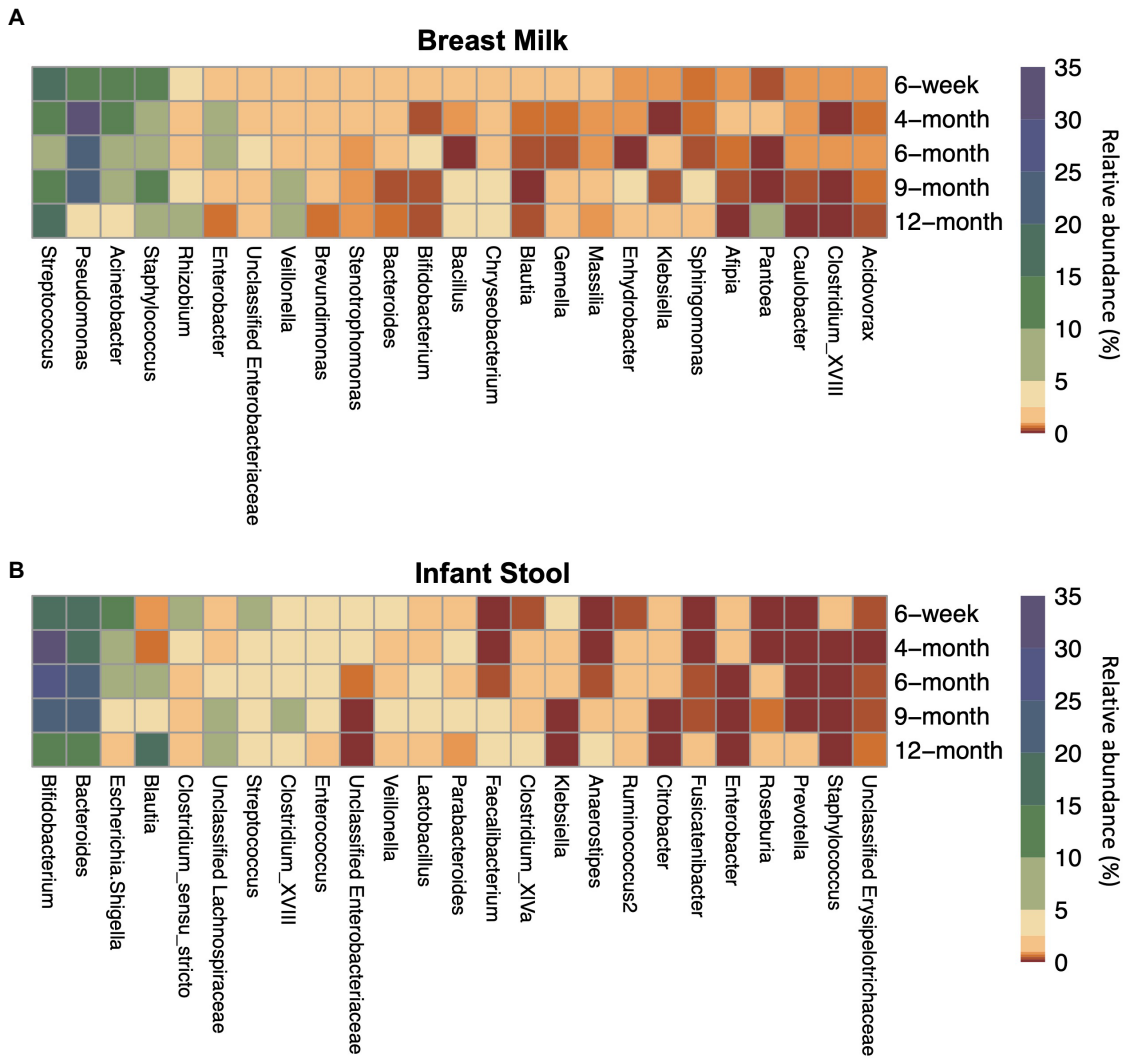
Top 25 most abundant taxa on average by sample type and timepoint for **(A)** breast milk and **(B)** infant stool. Colors indicate the average relative abundance of each taxon. The y-axis indicates the sample collection timepoint. For the x-axis, G indicates the genus while F indicates the family.

Across all collection time points, the most abundant taxon in milk (*n* = 245) was the genus *Streptococcus* (16.0%), followed closely by the genera *Pseudomonas* (15.8%), *Acinetobacter* (11.8%), and *Staphylococcus* (10.3%). *Streptococcus* was the most abundant genus at the 6-week (*n* = 181) and 12-month timepoints (*n* = 7), while at the 4-(*n* = 26), 6- (*n* = 16), and 9-month (*n* = 15) timepoints, *Pseudomonas* was the most abundant genus measured in breast milk. Most taxa had relatively consistent abundances from 6-weeks through 12-months of lactation (Figure 1; Supplementary Table S3).

The taxa in the infant gut, however, changed in abundance to a greater degree across the first year of life (Figure 1; Supplementary Table S3). From 6 weeks through 9 months (n_6 weeks_ = 151, n_4 months_ = 21, n_6 months_ = 20, n_9 months_ = 22), the two most abundant taxa in the infant gut were the genera *Bifidobacterium* (18.6–29.4%) and *Bacteroides* (19.5–25.0%). *Blautia* and *Lachnospiraceae* are present at less than 1% and around 2% of the infant gut microbiome, respectively, until 4-months of life, but begin to rise in abundance by 6 months. At 12 months (*n* = 113), *Blautia* was the most abundant taxon in the infant gut at 17.3%, followed by *Bacteroides* (13.4%), *Bifidobacterium* (13.3%), and the family *Lachnospiraceae* (9.93%).

### 3.3. Alpha diversity over the first year of life

**Figure 2:**
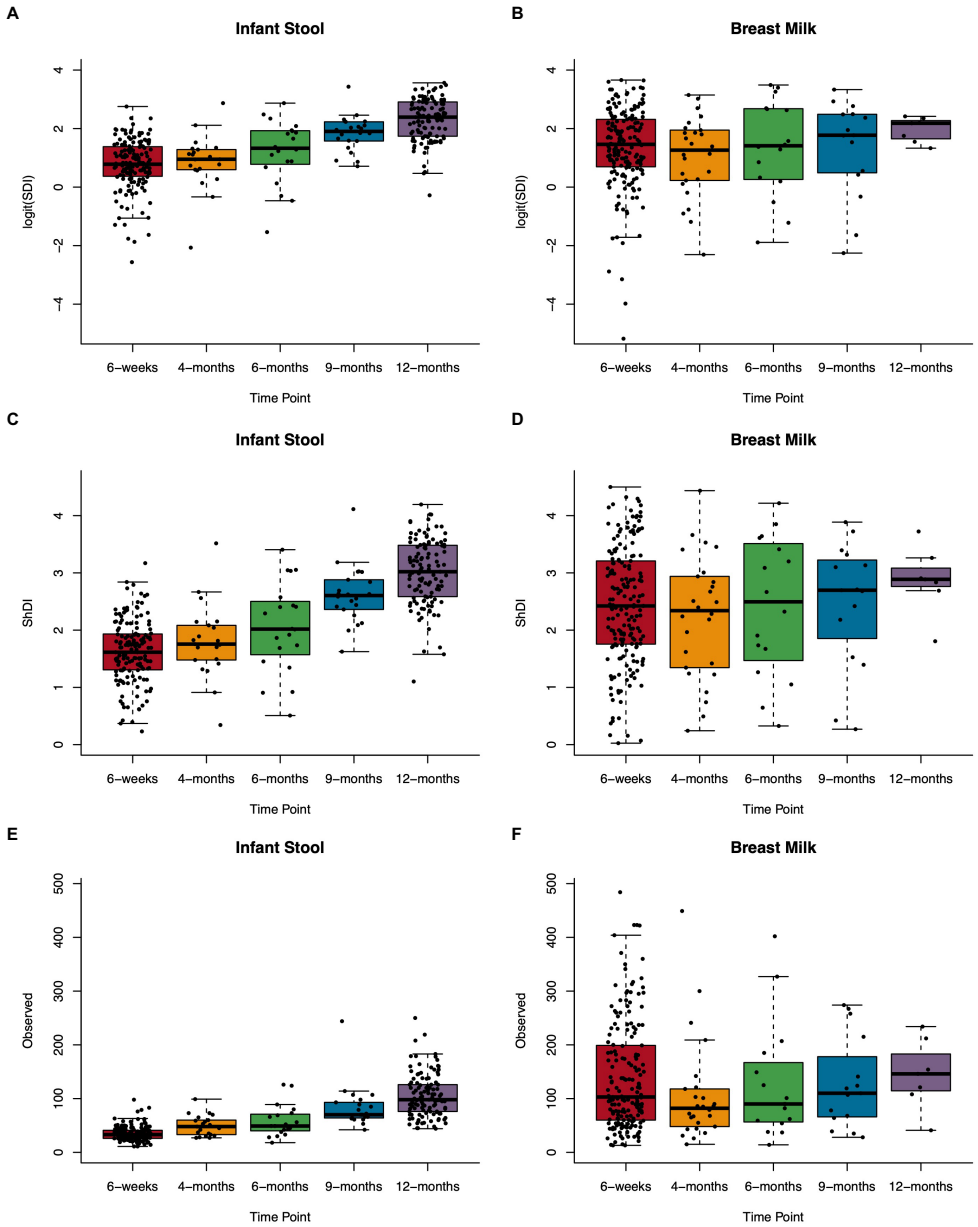
Microbial alpha diversity in the infant gut and human milk over the first year of life. Boxplots showing Simpson’s diversity index in **(A)** infant stool and **(B)** breast milk, Shannon diversity index in **(C)** infant stool and **(D)** breast milk, and observed ASVs in **(E)** infant stool and **(F)** breast milk from 6-weeks to 12-months of age.

We assessed several metrics of alpha diversity for the infant gut and human milk: SDI, ShDI, and the number of unique ASVs observed in a sample. In infant stool, all diversity metrics increased over the sample timepoints from 6 weeks through 12 months of life (Figure 2; Supplementary Table S4), while alpha diversity in human milk did not change over time. When we modeled the relation between age at sample collection and alpha diversity metrics using a linear model with a random effect for subject (Supplementary Figure S4), we found that every 100 days of life related to an increase in logit(SDI) of 0.47 (*n* = 327, value of *p* = 1.3 × 10^−42^), ShDI of 0.41 (*n* = 327, value of *p* = 4.3 × 10^−59^), and observed ASVs of 20.78 (*n* = 327, value of *p* = 3.9 × 10^−61^). These results did not differ when analyzing the rarefied data (Supplementary Figure S5; Supplementary Table S4).

### 3.4. Overlap between infant gut microbiota and breast milk microbiota in maternal–infant dyads

**Figure 3:**
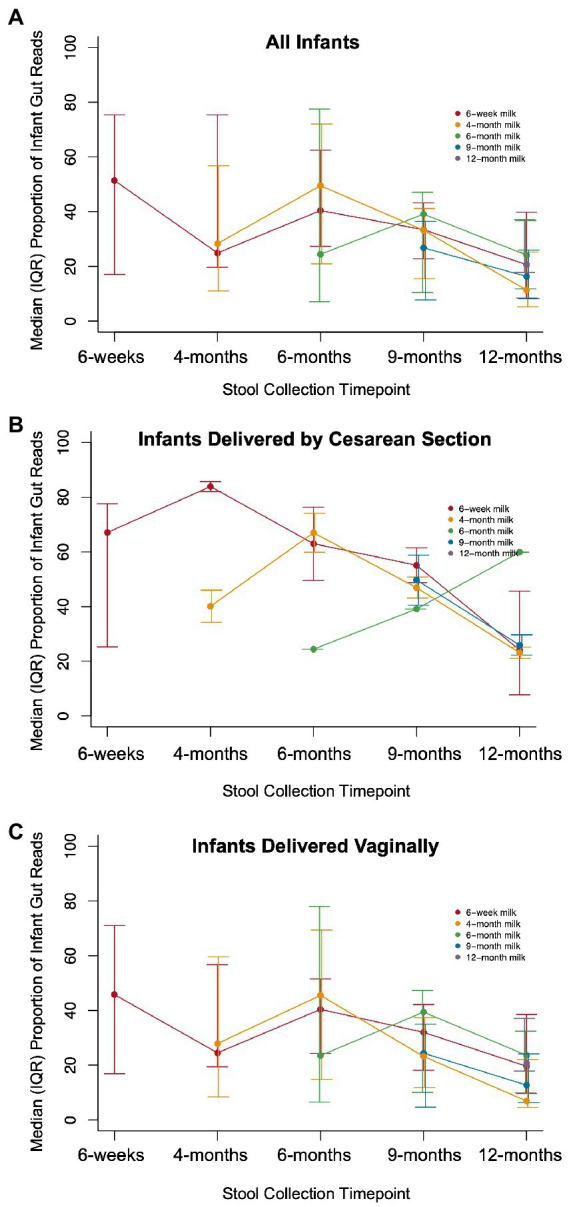
Proportion of infant gut reads from ASVs in paired milk over time. The median (IQR) of the total number of infant gut microbial reads from ASVs that also occurred in breast milk from an infant’s mother divided by the total number of microbial reads in a sample for **(A)** all infants (Kolmogorov–Smirnov test value of *p* = 0.0058 compared to random dyads), **(B)** infants delivered by Cesarean section (Kolmogorov–Smirnov test value of *p* = 0.054 compared to random dyads), and **(C)** infants delivered vaginally (Kolmogorov–Smirnov value of *p* = 0.49 compared to random dyads).

There were 144 maternal–infant dyads with milk and infant stool collected at 6 weeks postpartum, and 108 dyads with 12-month stool. Additional milk and infant stool samples were collected at intermediate timepoints across the first year of life, ranging from 5 to 19 dyads (Supplementary Table S1). We calculated the proportion of reads in the infant gut that were attributed to ASVs that were also detected in milk from the infant’s mother. The median proportion of infant stool reads attributable to 6-week breast milk for true mother-infant dyads was ~50% at 6-weeks and significantly higher compared to random mother-infant dyads (~25%, *n* = 144 dyads, Kolmogorov–Smirnov test value of *p* = 0.0058; Figure 3A; Table 2). Infants delivered by Cesarean section had a greater median proportion of reads from ASVs in breast milk consistently over the first year of life, with ~67% at 6 weeks (*n* = 40 dyads, Kolmogorov–Smirnov test value of *p* = 0.054 compared to random mother-infant dyads; Figure 3B; Table 2) compared to ~45% for infants delivered vaginally (*n* = 104 dyads, Kolmogorov–Smirnov value of *p* = 0.49 compared to random mother-infant dyads; Figure 3C; Table 2), a difference which was significant over the first year of life (*n* = 437 dyads, Coefficient_Cesarean Section_ = 9.5, Kenward-Roger value of *p* = 0.022; Supplementary Table S5). Additionally, the median proportion of infant gut reads from breast milk ASVs was ~65% for boys (Kolmogorov–Smirnov value of *p* = 0.0023 compared to random mother-infant dyads, Supplementary Figure S6A; Table 2), while in girls was only ~35% (Kolmogorov–Smirnov value of *p* = 0.95 compared to random mother-infant dyads, Table 2), and was consistently higher over the first year of life compared to girl infants (*n* = 437 dyads, Coefficient_Male_ = 9.4, Kenward-Roger value of *p* = 0.0088; Supplementary Figure S6B; Supplementary Table S5). Additionally, feeding a combination of breast milk and formula was associated with a lower proportion of infant gut reads from breast milk over the first year of life (*n* = 397, Coefficient_Combination feeding_ = −13.4, Kenward-Roger value of *p* = 1.7 × 10^−5^; Supplementary Table S5). However, an adjusted model including delivery mode, exposure to formula, infant sex, and age of the infant at sample collection only show significant effects of male sex and age of the infant at stool sample collection, and a marginally significant effect of Cesarean delivery (Supplementary Table S5). Excluding the 6-week timepoint, there appeared to be a greater proportion of reads from ASVs present in milk from previous rather than from contemporaneous collection timepoints (Figure 3).

**Table 2:** The overlap proportion of infant gut reads with breast milk ASVs at 6-weeks of age is greater for true pairs than for random pairs.

Group	*N*	*p*-value^a^
All infants	144	0.0058
Unexposed to formula	108	0.25
Vaginal	104	0.49
Cesarean	40	0.054
Vaginal, Unexposed to formula	82	0.35
Cesarean, Unexposed to formula	26	0.042
Males	78	0.0023
Females	66	0.95

a Calculated using the Kolmogorov–Smirnov test comparing the distribution of the overlap proportions for true mother-infant dyads compared to that for random mother-infant dyads.

### 3.5. Clustering analysis of infant gut and breast milk microbiota

To identify infant gut microbiome types (IGMTs) and breast milk microbiome types (BMTs) we used partitioning around medoids and assessed clustering across timepoints for infant stool and human milk. We identified six IGMTs from samples collected between 6-weeks and 12-months of age (Supplementary Figure S7), which were associated with the collection timepoint (Supplementary Table S6; *n* = 327, Fisher’s exact value of *p* = 0.00050). Most 12-month samples clustered in IGMT1 (36.3% of 12-month samples) and IGMT3 (46.9% of 12-month samples), while IGMTs 2 (81.4% of samples in cluster were 6-week through 9-month samples) and 4–6 (93.0, 97.5, and 98.1% of samples in each cluster, respectively, were 6-week through 9-month samples) were made up primarily of 6-week through 9-month samples (Supplementary Table S5). IGMT1 and IGMT3 had a higher proportion of *Blautia* and *Lachnospiraceae* compared to the other clusters, and IGMT1 had a lower abundance of *Bacteroides* compared to IGMT3. IGMT2 was characterized by a high abundance of *Bifidobacterium*, IGMT4 by high *Bacteroides*, IGMT5 by both *Bifidobacterium* and *Bacteroides*, and IGMT6 by a predominance of *Escherichia/Shigella*, *Clostridium*, and low diversity.

**Figure 4:**
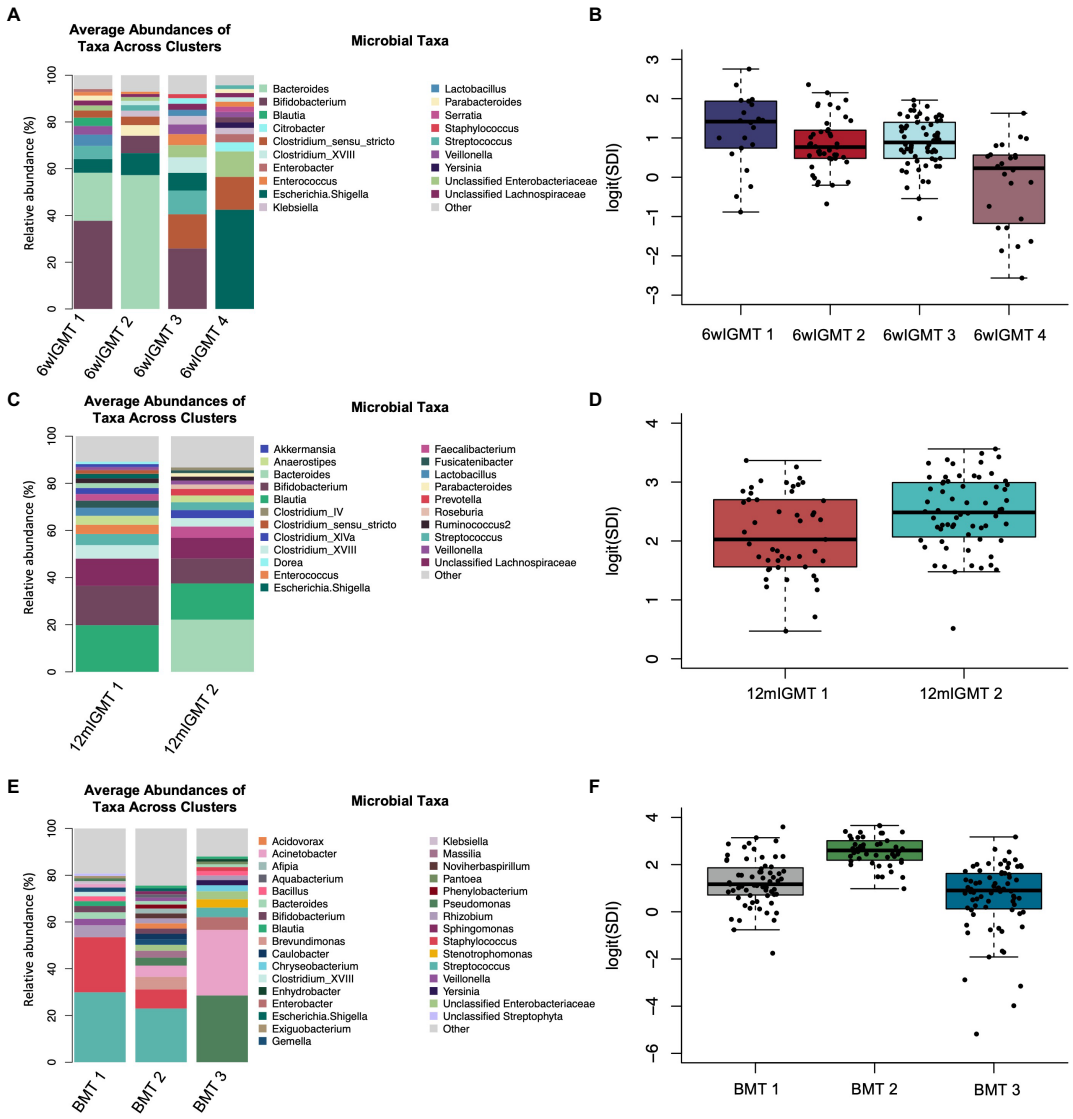
Infant gut and human milk microbiome clusters. **(A)** Barplot of the average relative abundance of microbial taxa overall in 6-week infant stool and by 6-week infant gut microbiome type (IGMT). **(B)** Boxplot of logit(Simpson’s diversity index) by 6-week IGMT (Kruskal-Wallis rank sum test, value of *p* = 1.6 × 10^−5^). **(C)** Barplot of the average relative abundance of microbial taxa overall in 12-month infant stool and by 12-month IGMT. **(D)** Boxplot of logit(Simpson’s diversity index) by 12-month IGMT (Kruskal-Wallis rank sum test, value of *p* = 0.0056). **(E)** Barplot of the average relative abundance of microbial taxa overall in 6-week breast milk and by 6-week breast milk microbiome type (BMT). **(F)** Boxplot of logit(Simpson’s diversity index) by 6wBMT (Kruskal-Wallis value of *p* = 3.3 × 10^−16^).

We also clustered 6-week infant stool samples and 12-month infant stool samples separately to identify 6wIGMTs and 12mIGMTs in order to prevent empty clusters when assessing the association between breast milk microbiome clusters and infant gut microbiome clusters. Within the 6-week stool samples, we identified four 6wIGMTs using partitioning around medoids (*n* = 151; Figure 4A; Supplementary Figures S8A,B), which were similar in composition to IGMTs 2 and 4–6 from the clustering performed on all samples. Simpson’s Diversity Index was lowest in 6wIGMT4, highest in 6wIGMT1, and intermediate in 6wIGMTs 2 and 3 (*n* = 151, Kruskal-Wallis rank sum test, value of *p* = 1.6 × 10^−5^; Figure 4B). Within 12-month samples (*n* = 113), two 12mIGMTs were identified and these clusters had similar composition to IGMTs 1 and 3 from the clustering performed on all samples (Figure 4C; Supplementary Figures S8C,D). Simpson’s diversity in 12mIGMT2 was higher than that in 12mIGMT1 (*n* = 113, Kruskal-Wallis rank sum test, value of *p* = 0.0056; Figure 4D).

In 6-week human milk samples (*n* = 181), we identified three breast milk microbiome types (BMTs) communities (Figure 4E; Supplementary Figures S8E,F). 6wBMT1 was characterized by a high abundance of *Streptococcus* and/or *Staphylococcus* and relatively moderate diversity, BMT2 by presence of *Streptococcus* and relatively high diversity, and BMT3 by high abundance of *Pseudomonas* or *Acinetobacter* along with lower diversity (*n* = 181, Simpson’s diversity Kruskal-Wallis value of *p* = 3.3 × 10^−16^; Figure 4F).

### 3.6. Changes in cluster membership for subjects with longitudinal samples collected

In order to assess the stability of the breast milk microbiome over time, we performed PAM clustering of all breast milk microbiome samples collected from subjects over the first year (*n* = 245) and obtained the BMT (Supplementary Figure S10). Twenty-five subjects had at least two samples of milk collected over time, contributing a total of 81 milk samples. 36% of subjects had all samples classified to the same cluster, while 44% had at least one sample classified to one different cluster than their first sample, and the remaining 20% of subjects had samples classified to all three BMTs. In univariate analyzes, the only variable associated with the degree of BMT cluster switching was the number of days postpartum the last milk sample was collected (Supplementary Table S7). A multivariate analysis using multinomial logistic regression showed that in addition to the number of days postpartum of collection of the last sample, antibiotic exposure during pregnancy was marginally associated with the degree of cluster switching, however the confidence intervals were very wide (Table 3; Supplementary Figure S11). Additionally, GUniFrac distances, a measure of community dissimilarity, between milk samples from the same woman were lower than those between milk samples from different women (*n* = 25, Kruskal-Wallis value of *p* = 4.38 × 10^−9^; Supplementary Figure S12A).

**Table 3:** Maternal variables and time elapsed associate with the stability of breast milk microbiota and infant gut microbiota over time.

Breast Milk	OR (95% CI)^a^^,^^b^	
	Cluster switching degree 1	Clustering switching degree 2
Days postpartum of last sample collected	1.03 (1.00–1.07)	1.16 (1.11–1.22)
Maternal pre-pregnancy BMI	1.86 (0.83–4.17)	4.55 (2.22–9.36)
Maternal age	0.61 (0.32–1.15)	2.14 (1.05–4.36)
Prenatal antibiotics	915.08 (0.08–11,075,210)	1051930.57 (73.01–15,156,890,000)
^a^Model adjusted for days postpartum of last sample collected, maternal pre-pregnancy BMI, maternal age, and prenatal antibiotic exposure		
^b^No cluster switching as reference level		
Infant Stool	OR (95% CI)^a^^,^^b^	
	Cluster switching degree 1	Clustering switching degree 2
Infant age at last sample collection	0.98 (0.95–1.01)	1.07 (1.02–1.12)
Maternal age	0.68 (0.45–1.02)	0.75 (0.53–1.06)
Prenatal antibiotics	167.79 (1.70–16,598)	4.40 (1.04–185.50)

a Model adjusted for days postpartum of last sample collected, maternal age, and prenatal antibiotic exposure.

b No cluster switching as reference level.

We performed an analogous analysis in the infant stool samples over time, excluding 12-month samples since they cluster separately from 6-week to 9-month samples. 22.2% of 27 infants with longitudinal stool samples available remained in the same IGMT through 9 months of age, while 44.4% had one sample belong to a different cluster and 33.3% belonged to three or more IGMTs through 9 months of age. The average GUniFrac distances between infant stool samples from the same subjects was lower than that between infant stool samples from different subjects through the first 9 months of life (*n* = 27, Kruskal-Wallis value of *p* = 2.9 × 10^−10^; Supplementary Figure S12B). IGMT cluster-switching over the first year of life was not associated with the tested maternal and infant factors in univariate analyzes (Supplementary Table S8). However, a multinomial logistic regression model adjusting for the age of the infant at the last sample collection showed significant positive associations with degree of IGMT switching for maternal antibiotics taken during pregnancy, infant Cesarean section delivery, and parity, and a marginal negative association of increasing maternal age with cluster switching in addition to a significant effect of infant age at last sample collection (Table 3; Supplementary Figure S13).

### 3.7. Association of human milk microbiome patterns with infant gut microbiome patterns

**Figure 5:**
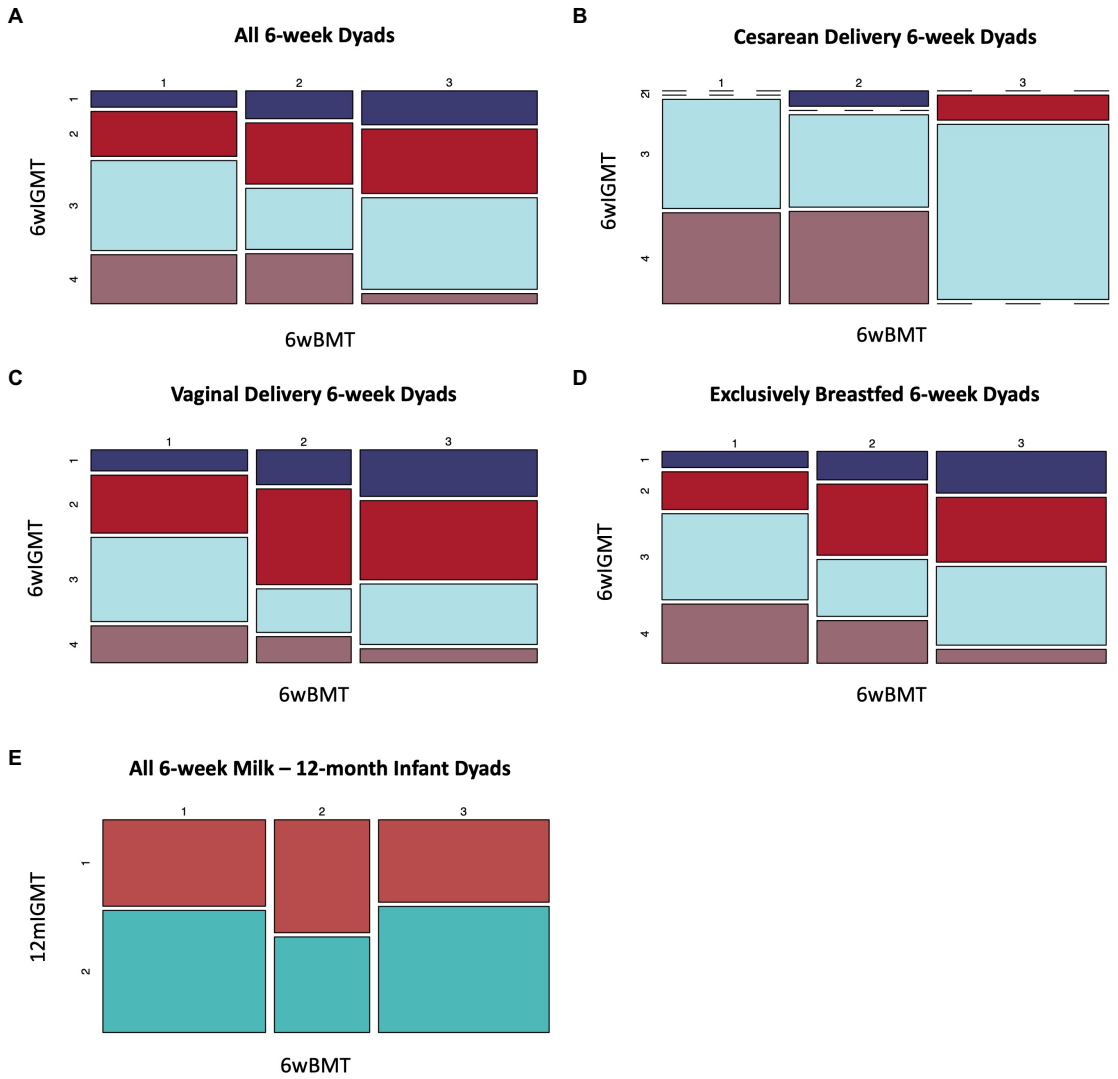
Breast milk microbiome type is associated with infant gut microbiome type at 6-weeks of age. **(A)** All dyads (*n* = 144, Fisher’s exact value of *p* = 0.039), **(B)** dyads where infants were delivered by Cesarean section (*n* = 40, Fisher’s exact value of *p* = 0.0028), **(C)** infants delivered vaginally For 6wBMT vs. 6wIGMT (*n* = 104, Fisher’s exact value of *p* = 0.29), **(D)** dyads with no formula exposure (*n* = 108, Fisher’s exact value of *p* = 0.080). **(E)** There was no association between 6wBMT and 12mIGMT (*n* = 108, Fisher’s exact value of *p* = 0.51).

We identified an association between BMT cluster membership at 6 weeks postpartum and 6wIGMT cluster membership (*n* = 144 dyads, Fisher’s exact test value of *p* = 0.039; Supplementary Table S9). Specifically, infants of mothers in BMT1 were less likely to belong to 6wIGMT1 or 2, whereas infants of mothers in BMT2 were less likely to belong to 6wIGMT3, and infants of mothers in BMT3 were less likely to belong to 6wIGMT4 (Figure 5A). When stratifying the analysis to infants delivered vaginally and by Cesarean section, the relation of milk and stool microbiome cluster membership was predominantly observed in infants delivered by Cesarean section. Infants delivered by Cesarean section mostly belong to 6wIGMT3 and 4, but no infants of mothers in BMT3 belonged to 6wIGMT4 (*n* = 40 dyads, Fisher’s exact test value of *p* = 0.0028; Figure 5B; Supplementary Table S9). The same pattern as in the unstratified analysis was observed in the vaginal delivery group (*n* = 104 dyads, Fisher’s exact test value of *p* = 0.29; Figure 5C; Supplementary Table S9), and in infants who were unexposed to formula (*n* = 108 dyads, Fisher’s exact test value of *p* = 0.080; Figure 5D; Supplementary Table S9), but these were not statistically significant. Additionally, 6wBMT was related to 6wIGMT in male infants (*n* = 78 dyads, Fisher’s exact test value of *p* = 0.020) but not in female infants (*n* = 66 dyads, Fisher’s exact test value of *p* = 0.51; Supplementary Figure S14). There was no association between the 6wBMT and 12mIGMT (*n* = 108 dyads, Fisher’s exact test value of *p* = 0.51; Figure 5E). We also observed an association between the most abundant breast milk microbe and the most abundant infant gut microbe at 6-weeks (*n* = 144 dyads, Fisher’s exact test *p*-vlaue = 0.036; Supplementary Table S10).

### 3.8. Relation between milk and infant gut microbial community structures

We used the Mantel test to assess the association between milk and infant gut microbial community structures as characterized by ordination of samples using GUniFrac distances and controlled for important covariates using a third distance matrix based on maternal pre-pregnancy BMI, delivery mode, exposure to formula, exposure to antibiotics during pregnancy, maternal age, and infant sex. Overall, there was no significant cross-sectional correlation between microbial community structures (*n* = 113 dyads, Mantel test *Z*-statistic = −0.024, value of *p* = 0.77; Supplementary Table S11), however milk microbial communities were related to infant gut microbial communities longitudinally. For example, 6-week breast milk microbial communities were related to 6-month infant gut microbial communities (*n* = 12 dyads, Mantel test *Z*-statistic = 0.53, value of *p* = 0.001), 4-month breast milk to 6-month infant stool (*n* = 15 dyads, Mantel test *Z*-statistic = 0.21, value of *p* = 0.041), and 6-month breast milk to 9-month infant stool (*n* = 13 dyads, Mantel test *Z*-statistic = 0.34, value of *p* = 0.023). Among infants that were unexposed to formula at the time of stool collection (Supplementary Table S12), a similar trend was observed; associations were identified between 6-week breast milk microbial communities and 6-month infant gut microbial communities (*n* = 10 dyads, Mantel test *Z*-statistic = 0.48, value of *p* = 0.004), 6-month breast milk and 9-month infant gut (*n* = 10 dyads, Mantel test *Z*-statistic = 0.38, value of *p* = 0.022), and 9-month breast milk and 9-month infant gut microbial communities (*n* = 12 dyads, Mantel test *Z*-statistic = 0.27, value of *p* = 0.03). The same trend was observed in vaginally delivered infants (Supplementary Table S13); there were not enough maternal–infant pairs at non-6-week timepoints to assess these associations in infants delivered by Cesarean section or stratified by infant sex. In female infants, there was a marginal association between the 6-week breast milk microbiome and the 12-month infant gut microbiome (*n* = 38 dyads, Mantel test *Z*-statistic = 0.12, value of *p* = 0.059).

### 3.9. Correlation between microbial taxa in milk and in the infant gut

**Figure 6:**
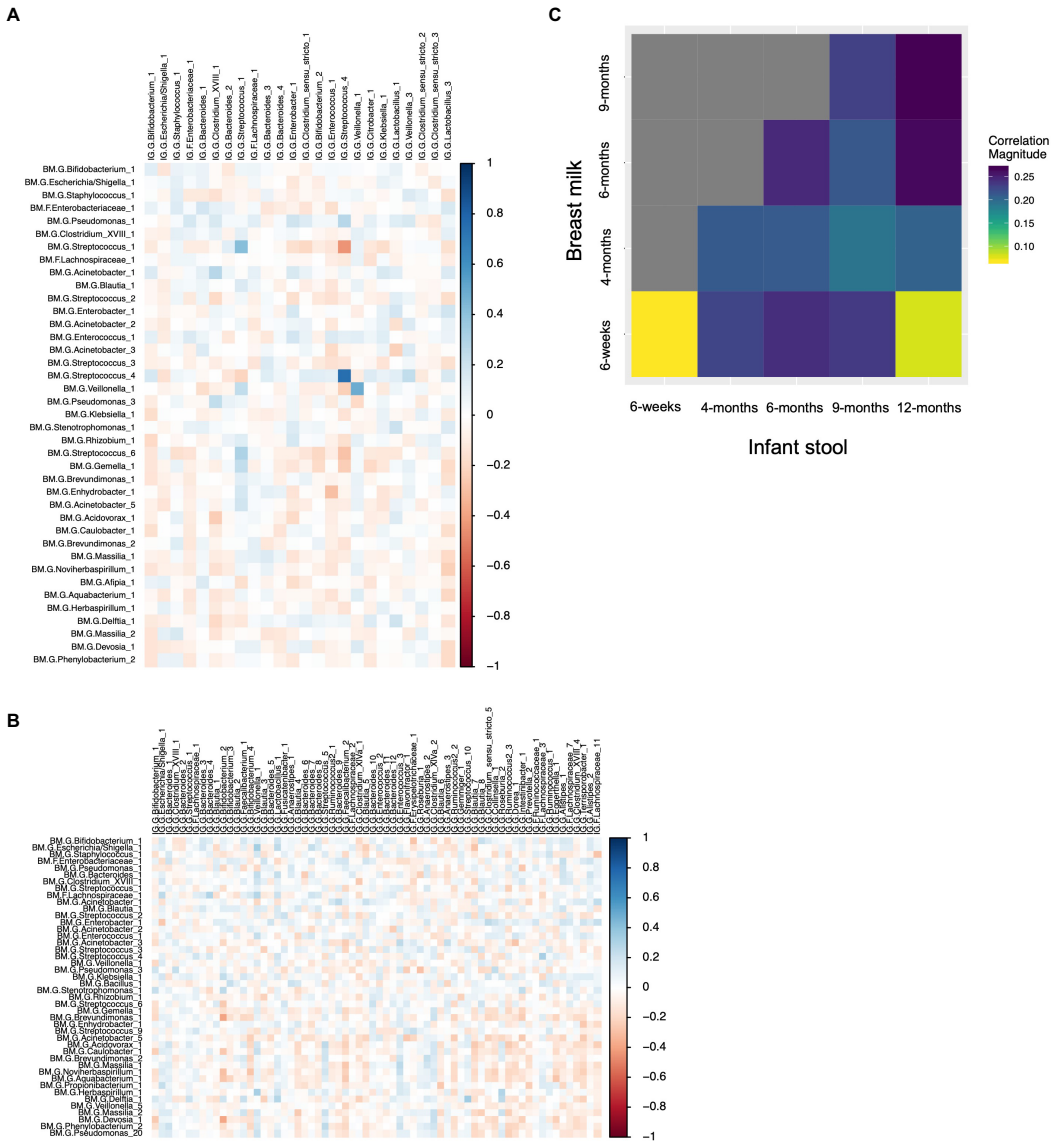
Correlations between breast milk and infant gut ASVs occur between both the same and different ASVs, and are stronger overall longitudinally. **(A)** Spearman correlation between 6-week breast milk microbial taxa and 6-week infant gut microbial taxa. **(B)** Spearman correlation between 6-week breast milk microbial taxa and 12-month infant gut microbial taxa. **(C)** Correlation plot showing the overall magnitude of microbial taxa correlation for each breast milk–infant gut timepoint combination.

We computed the Spearman correlation between relative abundances of ASVs in milk and in the infant gut across the first year of life. The overall magnitude of correlation was lowest between 6-week milk and 6-week stool (*n* = 144 dyads, average rho = 0.074) and was higher between milk and stool samples collected at subsequent time points (for example, *n* = 14 dyads, average rho = 0.24, 6-week breast milk vs. 6-month stool; Figure 6). Correlation between milk and infant gut ASVs at cross-sectional timepoints was higher for milk collected at 4 months and after. Statistically significant correlations of taxa in milk and infant stool at the 6-week timepoint (*n* = 144 dyads) were observed between the same ASV as well as between different ASVs in milk and infant stool; for example *Streptococcus_4* (rho = 0.76, *q*-value = 5.8 × 10^−25^)*, Streptococcus_1* (rho = 0.42, *q*-value = 1.3 × 10^−7^)*, and Veillonella_1* (rho = 0.49, *q*-value = 6.5 × 10^−6^) in milk were positively associated with their abundance in infant stool, while breast milk *Streptococcus_1* was negatively related to infant gut *Streptococcus_4* (rho = −0.44, *q*-value = 2.5 × 10^−5^), breast milk *Enhydrobacter_1* was negatively associated with infant gut *Enterococcus_1* (rho = −0.30, *q*-value = 0.039), and breast milk *Acinetobacter_1* was positively associated with infant gut *Clostridium_XVIII_1* (rho = 0.28, *q*-value = 0.081; Table 4; Figure 6A). These associations were also observed at 6 weeks of age within delivery mode groups and among infants who were unexposed to formula at the time of sample collection (Supplementary Figure S15). There were also significant associations, both cross-sectionally and longitudinally, between later breast milk and infant gut timepoints (Supplementary Table S14). Notably, the abundance of *Pantoea_1* in milk was positively associated with *Lachnospiraceae* (three different varieties), observed between 4-month milk and 12-month infant stool (*n* = 18 dyads) and between 6-month milk and 9-month stool (*n* = 15 dyads; Supplementary Table S14).

**Table 4:** Taxa in breast milk and infant stool associate with one another.

6-week milk vs. 6-week stool (*n* = 144 dyads)				
Breast milk taxon	Infant gut taxon	rho	*p*-value	*q*-value
*Streptococcus_4*	*Streptococcus_4*	0.76	7.3E-28	5.8E-25
*Streptococcus_1*	*Streptococcus_1*	0.42	3.2E-10	1.3E-07
*Veillonella_1*	*Veillonella_1*	0.49	2.4E-08	6.5E-06
*Streptococcus_1*	*Streptococcus_4*	−0.44	1.2E-07	2.5E-05
*Streptococcus_6*	*Streptococcus_1*	0.31	1.7E-04	0.027
*Enhydrobacter_1*	*Enterococcus_1*	−0.30	2.9E-04	0.039
*Streptococcus_6*	*Streptococcus_4*	−0.29	4.9E-04	0.056
*Acinetobacter_1*	*Clostridium_XVIII_1*	0.28	8.1E-04	0.081
*Pseudomonas_1*	*Streptococcus_4*	0.27	0.00104	0.092
6-week milk vs. 9-month stool (*n* = 16 dyads)				
Breast milk taxon	Infant gut taxon	rho	*p*-value	*q*-value
*Stenotrophomonas_1*	*Lachnospiraceae_19*	0.85	3.7E-05	0.056
*Bacteroides_4*	*Akkermansia_1*	0.84	3.9E-05	0.056
6-week milk vs. 12-month stool (*n* = 108)				
Breast milk taxon	Infant gut taxon	rho	*p*-value	*q*-value
*Brevundimonas_1*	*Bifidobacterium_2*	−0.399	1.9E-05	0.050
*Devosia_1*	*Bifidobacterium_2*	−0.387	3.6E-05	0.050

## 4. Discussion

We measured microbial communities in breast milk and infant stool from 189 mother-infant dyads across the first year of life using targeted sequencing of the 16S rRNA gene. Clustering of infant gut microbial communities revealed four 6wIGMTs, separated mostly on abundances of *Bacteroides*, *Bifidobacterium*, *Clostridium*, *Streptococcus*, and *Escherichia*/*Shigella*. Three BMTs were also identified, characterized by abundance of *Streptococcus*, *Staphylococcus*, *Acinetobacter*, *Pseudomonas*, and microbial diversity. At 6-weeks postpartum, BMT was associated with 6wIGMT, but there was no association between the 6wBMT and the 12mIGMT. This may be due to the fact that by 12 months of age, infants consume a variety of solid foods and no longer rely on breast milk as their main source of nutrition (Grummer-Strawn et al., 2008) which likely drives the increase in microbes such as *Bifidobacterium* and *Bacteroidetes* (Fallani et al., 2011; Koenig et al., 2011); thus as the gut microbiome begins to mature and look more adult-like (Backhed et al., 2015) and in our study 12mIGMTs separated only by the most abundant bacteria, it is not unexpected that the association of breast milk microbial communities with the infant gut microbiome would be too subtle to detect using this method.

Our results also suggest that the effect of breast milk microbial communities on the infant gut microbiome may be modified by multiple factors. For example, in 6-week cross-sectional analyzes stratified by delivery mode, the effect of the 6wBMT on the 6wIGMT was primarily observed in infants delivered by Cesarean section, although a similar pattern was also observed in vaginally delivered infants. Additionally, infants delivered by Cesarean section had greater proportions of microbial reads from ASVs that were present in the breast milk they consumed compared to infants who were delivered vaginally, which is in line with the recent finding that while infants delivered by Cesarean section have a reduced colonization by maternal stool microbiota, their colonization by breast milk microbiota is higher than vaginally delivered infants (Bogaert et al., 2023). Previous research indicates that the intestinal microbiome of infants delivered by Cesarean section persistently differs significantly from that of infants delivered vaginally, most profoundly in a depletion of *Bacteroides* (Dominguez-Bello et al., 2010; Azad et al., 2013; Jakobsson et al., 2014; Backhed et al., 2015; Madan et al., 2016) past the first week of life (Shao et al., 2019; Mitchell et al., 2020; Bogaert et al., 2023) as well as a decrease in microbial diversity (Jakobsson et al., 2014; Bokulich et al., 2016), which in theory could cause this group to be more susceptible to colonization by the pre-and probiotics derived from breast milk (Grönberg et al., 1992; Thurl et al., 2010; Jost et al., 2013). Similarly, we observed a greater effect of BMT of 6wIGMT in male infants and also a greater proportion of infant gut microbial reads attributable to ASVs in milk compared to female infants. It is possible that male infants’ gut microbiomes are more strongly associated with breast milk microbiota due to their lower bacterial diversity, lower abundance of *Clostridiales*, and higher abundance of *Enterobacteriales* compared to female infants (Cong et al., 2016). Additionally, it is well established that the infant male gut microbiome is more greatly affected by exposures than that of females, for example, the effects of early prenatal stress (Jasarevic et al., 2017) and arsenic (Hoen et al., 2018) exposure are primarily observed in males. Our study suggests that the association of the gut microbiome in male infants and those delivered by Cesarean section, both of which are known to have less diverse gut microbiomes, is stronger than in female infants and infants delivered vaginally.

When assessing the associations between human milk microbiota and infant gut microbiota using GUniFrac distances in milk and infant stool, we observed that the effect of breast milk microbes on the infant gut microbiome may be somewhat delayed. For example, the microbial community structure of 6-week breast milk was not related the that of the infant gut at 6 weeks, but was related to the microbiome of the infant gut at 6 months of age and between 6-month breast milk and 9-month infant stool. The same pattern was observed at other longitudinal timepoint pairs and within infants who were unexposed to formula at the time of stool collection and vaginally delivered infants. However, due to limited sample sizes at intermediate time points, further examination in larger studies and perhaps experimentally in animal models is warranted in order to determine whether and to what extent there is a time delay in the association of breast milk microbes with infant gut microbes.

In our study, we observed correlations between individual ASVs in breast milk and infant stool both between the same shared ASV as well as between different microbial taxa. Some of the associations we identified are consistent with what others have reported, such as the positive correlations of different *Streptococcus* ASVs with themselves and negative correlations between different *Streptococcus* ASVs in breast milk and the infant gut in early life (Fehr et al., 2020), while the associations we identified between different taxa in human milk and the infant gut are novel. This observation suggests that in addition to possible sharing of microbes between breast milk and the infant gut *via* ingestion of breast milk by infants and inoculation of breast tissue with infant oral microbes, these complex microbial communities may interact with one another indirectly. Several possible explanations for these indirect effects exist, such as differences in fatty acids (Kumar et al., 2016), human milk oligosaccharides (Aakko et al., 2017; Cabrera-Rubio et al., 2019), micronutrients (Gomez-Gallego et al., 2018), or other metabolites which could be influenced or produced by microbiota. Future studies will be necessary to assess the extent to which these explain associations between breast milk microbiota and infant gut microbiota.

We also assessed the stability of the breast milk and infant gut microbiomes over the first year of life by examining the frequency of subjects switching between BMTs and IGMTs, and the change in the most abundant taxa. Overwhelmingly, breast milk microbial profiles were not stable within subjects. This is in contrast to previous evidence suggesting that milk microbes in women are stable in mature milk over time, however, samples in that study were collected over only 4 weeks, representing a fairly short-term assessment (Hunt et al., 2011). In our study, samples were collected at up to five timepoints across 1 year, and the minimum time between milk sample collection for a given subject was 8 weeks. In fact, we found that the later that the last breast milk sample from a subject was collected, the probability of a subject having a longitudinally collected sample classified to a different breast milk cluster increased. This suggests that collecting multiple longitudinal breast milk samples over the first year of an infant’s life may be necessary for accurately characterizing an infant’s early life microbial exposures from breastfeeding. We also found evidence that exposure to antibiotics during pregnancy increased the probability of the breast milk microbiome changing over time. It will be important to continue to study how the microbial communities in breast milk change over time within an individual, the factors that influence those changes, and their effect on the infant gut microbiome and infant developmental outcomes by collecting samples more frequently and over a longer period of time in future studies.

There was also a large degree of change in the infant gut microbiome over time in terms of switching between IGMTs and changes in the most abundant taxa. In addition to an older infant age at the time of the last stool collection, likely reflecting the normal microbiome maturation across the first year of life, delivery by Cesarean section was positively associated with the degree of IGMT cluster switching. Maternal exposure to antibiotics during pregnancy and higher parity also increased the probability of an infant belonging to two or more IGMTs up to 9 months of age. Additional study is necessary in order to clarify the clinical implications of this microbial variation in the infant gut. Ultimately, however, GUniFrac distances within a subject for both the human milk microbiome and the infant gut microbiome were lower than distances between subjects for longitudinally collected samples. This may be in part due to the fact that the GUniFrac distance metric applies a weight to capture differences between samples in both highly abundant and rare microbes, while BMT and IGMT clusters largely reflect differences in more highly abundant taxa. It is therefore possible that the microbial taxa with a lower abundance overall in the infant gut may be more stable within individuals.

Our study had several key strengths, including a large overall sample size and collection of both cross-sectional and longitudinal milk and infant stool samples over the first year of life. Additionally, our study population originates from a single cohort. However, the sample sizes for maternal–infant dyads at intermediate timepoints were small, reducing the ability to thoroughly assess potential confounding factors and limiting statistical power to detect associations; however, these timepoints were largely restricted to vaginally delivered infants as a feature of the study design. When possible, we adjusted analyzes for the most important potential confounders and tested associations stratified by delivery mode, infant sex, and restricted to infants unexposed to formula. Additionally, the usage of 16S rRNA sequencing did not allow for determination of microbial species or strains, and the observational nature of the study cannot assess whether the co-occurrence of microbes and correlations between microbial taxa in breast milk and the infant gut are due to colonization of the infant gut by breast milk microbiota or rather that both the milk and infant gut microbiomes vary together in response to some other factor such as the infant oral microbiome, for which data is unavailable.

## 5. Conclusion

In conclusion, we identified an association between the breast milk microbiome profile and the infant microbiome profile at 6-weeks through clustering analysis that was primarily observed in infants delivered by Cesarean section. Additionally, specific microbial taxa in milk were correlated with those in the infant gut both in contemporaneously and longitudinally collected samples, occurring both between the same taxa in milk and infant stool as well as between different microbial taxa. These results suggest that the breast milk microbiome may additionally influence the infant gut microbiome through complex molecular interactions such as through metabolite signaling. Additionally, we identified factors in additional to time which appear to influence the stability of breast milk and infant gut microbiota over time, including maternal exposure to antibiotics during pregnancy.

## Data availability statement

Publicly available datasets were analyzed in this study. This data can be found here: Sequence Read Archive under the accession number PRJNA296814 (https://www.ncbi.nlm.nih.gov/bioproject/PRJNA296814/).

## Ethics statement

The studies involving human participants were reviewed and approved by Center for the Protection of Human Subjects at Dartmouth. Written informed consent to participate in this study was provided by the participants’ legal guardian/next of kin.

## Author contributions

SL, JM, MK, HM, BC, and AH designed the study. HM performed the experiments. SL analyzed the data. SL, JM, MK, HM, BC, and AH wrote the manuscript. All authors contributed to the article and approved the submitted version.

## Funding

This study was by supported by funding from the National Institutes of Health (grants NIGMS P20GM104416, NIEHS P01ES022832, NIEHS P42ES007373, NLM K01LM011985, NLM R01LM012723, NCI R01CA216265, and NIDCR R01DE022772), the US Environmental Protection Agency (RD83544201), and the Rosaline Borison Memorial Fund.

## Conflict of interest

SL conducted this research while at the Geisel School of Medicine at Dartmouth and is currently affiliated with Nightingale Health Plc.

The remaining authors declare that the research was conducted in the absence of any commercial or financial relationships that could be construed as a potential conflict of interest.

## Publisher’s note

All claims expressed in this article are solely those of the authors and do not necessarily represent those of their affiliated organizations, or those of the publisher, the editors and the reviewers. Any product that may be evaluated in this article, or claim that may be made by its manufacturer, is not guaranteed or endorsed by the publisher.

## Abbreviations

BMT: Breast milk microbiome type
IGMT: infant gut microbiome type
GUniFrac: generalized UniFrac
SDI: Simpson’s Diversity Index
ShDI: Shannon Diversity Index
ASVs: amplicon sequence variants
NHBCS: New Hampshire Birth Cohort Study
MBL: Marine Biological Laboratory
PAM: partitioning around medoids
PCoA: principal coordinates analysis
BMI: body mass index
