# Supplementary material for: Human milk-associated bacterial communities associate with the infant gut microbiome over the first year of life
Source: Front Microbiol. 2023 Apr 17;14:1164553. doi: 10.3389/fmicb.2023.1164553 (PMC10149717; doi:10.3389/fmicb.2023.1164553)
Supplement: Supplementary file 1 [file Data_Sheet_1.PDF]

Supplementary Material

Human Milk-associated Bacterial Communities Associate with the Infant Gut Microbiome Over the First Year of Life

Sara N. Lundgren[1,2], Juliette C. Madan[1,3,4], Margaret R. Karagas[1,5], Hilary G. Morrison[6], Brock C. Christensen[1,5,7*#], Anne G. Hoen[1,8,9#]

*** Correspondence:** Brock C. Christensen: Brock.C.Christensen@dartmouth.edu

1 Supplementary Figures and Tables

1.1 Supplementary Figures

Supplementary Table 1. Maternal-infant dyads for each timepoint combination.

		Infant stool collection timepoints				
		6 weeks	4 months	6 months	9 months	12 months
**Breast milk collection timepoints**	6 weeks	144	15	14	16	108
	4 months	-	17	16	19	18
	6 months	-	-	13	15	11
	9 months	-	-	-	14	12
	12 months	-	-	-	-	5

Supplementary Table 2. Proportion of infants receiving breast milk, infant formula, and solids at the time of each stool sample collection

	Breast milk only	Breast milk and formula only	Breast milk and solids	Breast milk, formula, and solids	Unknown type of milk and unknown if solids introduced	Unknown type of milk, solids introduced
**6 weeks**	72.9	11.3	1.5	0.8	13.5	0
**4 months**	63.2	0	15.8	10.5	10.5	0
**6 months**	10	5	50	30	5	0
**9 months**	0	0	59.1	40.9	0	0
**12 months**	0	0	25.5	60	13.6	0.9

Supplementary Table 3. Top most abundant microbial taxa in human milk and the infant gut over the first year of life.

Microbial taxa	Overall, Relative abundance (%)	Milk, Relative abundance (%)						Infant gut, Relative abundance (%)					
		Overall (n = 245)	6-week (n = 181)	4-month (n = 26)	6-month (n = 16)	9-month (n = 15)	12-month (n = 7)	Overall (n = 327)	6-week (n = 151)	4-month (n = 21)	6-month (n = 20)	9-month (n = 22)	12-month (n = 113)
G.Bifidobacterium	11.2	1.6	1.7	0.3	3.8	0.4	0.5	18.5	18.6	33.0	29.4	20.5	13.3
G.Bacteroides	11.0	1.6	1.8	1.1	2.0	0.4	0.6	18.0	19.5	19.6	25.0	22.9	13.4
G.Streptococcus	9.6	16.0	17.9	10.6	5.9	11.3	19.3	4.8	5.9	4.2	3.2	3.5	3.9
G.Pseudomonas	6.8	15.8	12.6	32.2	24.9	21.5	4.3	< .1 %	0.1	< .1 %	< .1 %	< .1 %	< .1 %
G.Acinetobacter	5.1	11.8	12.9	11.0	9.1	6.5	4.4	< .1 %	< .1 %	< .1 %	< .1 %	< .1 %	< .1 %
G.Escherichia.Shigella	4.7	0.7	0.7	< .1 %	3.0	0.2	0.2	7.7	13.4	7.3	6.0	3.8	1.3
G.Staphylococcus	4.7	10.3	11.0	8.3	7.1	10.1	6.7	0.5	1.0	0.1	< .1 %	< .1 %	< .1 %
G.Blautia	4.6	1.2	1.4	0.6	0.4	< .1 %	0.3	7.1	0.9	0.6	5.9	4.9	17.3
G.Clostridium_sensu_stricto	3.2	0.4	0.3	< .1 %	1.2	0.2	0.6	5.3	9.7	3.2	1.5	1.8	1.2
F.Lachnospiraceae	3.1	0.4	0.4	0.2	1.0	< .1 %	0.1	5.1	2.0	1.3	2.9	6.4	9.9
G.Clostridium_XVIII	2.7	0.8	0.9	< .1 %	0.9	0.2	0.1	4.1	3.6	4.7	3.8	5.0	4.5
F.Enterobacteriaceae	2.4	2.2	2.3	2.0	2.8	2.0	1.7	2.4	4.7	3.6	0.6	< .1 %	< .1 %
G.Veillonella	2.2	1.9	1.4	1.4	2.0	5.7	6.2	2.4	2.9	2.3	1.8	4.8	1.5
G.Enterococcus	1.7	0.5	0.4	0.5	1.7	< .1 %	0.1	2.5	2.8	3.2	2.8	2.7	2.0
G.Enterobacter	1.6	2.9	2.3	5.3	8.5	1.5	0.7	0.7	1.2	1.7	< .1 %	< .1 %	< .1 %
G.Lactobacillus	1.4	0.4	0.4	< .1 %	1.4	0.3	0.2	2.1	2.0	2.2	2.8	4.3	1.7
G.Rhizobium	1.3	3.0	3.1	1.8	1.9	2.6	8.3	< .1 %	< .1 %	< .1 %	< .1 %	< .1 %	< .1 %
G.Klebsiella	1.2	0.9	1.0	< .1 %	1.2	0.5	2.3	1.5	2.7	2.0	1.5	< .1 %	< .1 %
G.Parabacteroides	1.1	0.4	0.5	< .1 %	0.7	< .1 %	0.1	1.7	1.9	3.4	1.3	3.5	0.9
G.Faecalibacterium	1.0	0.1	0.2	< .1 %	0.1	< .1 %	< .1 %	1.6	< .1 %	< .1 %	0.3	3.3	4.0
G.Clostridium_XlVa	1.0	0.2	0.2	< .1 %	0.3	< .1 %	0.2	1.5	0.4	1.3	1.3	2.1	3.0
G.Anaerostipes	0.8	0.2	0.3	< .1 %	0.3	< .1 %	< .1 %	1.3	< .1 %	< .1 %	0.4	1.4	3.3
G.Brevundimonas	0.8	1.8	1.9	2.0	1.7	1.7	0.6	< .1 %	< .1 %	< .1 %	< .1 %	< .1 %	< .1 %
G.Stenotrophomonas	0.7	1.6	1.7	2.1	0.8	0.8	1.0	< .1 %	< .1 %	< .1 %	< .1 %	< .1 %	< .1 %
G.Citrobacter	0.7	0.2	0.3	< .1 %	0.2	< .1 %	0.2	1.0	1.9	1.3	1.5	< .1 %	< .1 %

[a]*G.* and *F.* indicate the level of taxonomy to be genus or family

Supplementary Table 4. Microbial alpha diversity increases in the first year of life in infant stool.

	Non-rarefied		Rarefied	
**Infant Gut Alpha Diversity**	**Coefficient[a]**	***p*-value[b]**	**Coefficient[a]**	***p*-value[b]**
Simpson's diversity	0.4664	1.25E-42	0.4625	8.40E-43
Shannon diversity	0.4115	4.32E-59	0.4009	2.54E-59
Observed ASVs	20.78	3.93E-61	12.45	4.14E-63

	Non-rarefied		Rarefied	
**Breast Milk Alpha Diversity**	**Coefficient[a]**	***p*-value[b]**	**Coefficient[a]**	***p*-value[b]**
Simpson's diversity	0.04430	0.67	0.0196	0.85
Shannon diversity	0.03371	0.65	0.018	0.81
Observed ASVs	-4.544	0.55	-1.622	0.64

[a]per 100 days

[b]Kenward-Roger approximation

Supplementary Table 5. Differences in proportion of infant gut microbial reads from ASVs in paired milk by infant factors.

	Unadjusted		Adjusted[a]	
**Variable**	**Coefficient**	***p*-value[b]**	**Coefficient**	**p-value[b]**
Combination feeding	-13.4	1.7E-05	1.0	0.77
Cesarean section	9.5	0.022	7.8	0.074
Male infants	9.400	0.0088	10.3	0.0064
Age of infant at stool sample (days)	-0.073	5.9E-18	-0.074	1.1E-11

[a]Model including combination feeding, Cesarean section, male infant sex, and age of infant at stool sample collection

[b]Kenward-Roger approximation

Supplementary Table 6. Overall infant gut microbiome types (IGMTs) are related to collection timepoint.

	6 weeks	4 months	6 months	9 months	12 months
**IGMT1**	1 (2.2)	0 (0.0)	1 (2.2)	3 (6.5)	41 (89.1)
**IGMT2**	41 (58.6)	7 (10.0)	5 (7.1)	4 (5.7)	13 (18.6)
**IGMT3**	1 (1.6)	0 (0.0)	4 (6.3)	5 (7.9)	53 (84.1)
**IGMT4**	41 (71.9)	5 (8.8)	3 (5.3)	4 (7.0)	4 (7.0)
**IGMT5**	21 (55.3)	5 (13.2)	5 (13.2)	6 (15.8)	1 (2.6)
**IGMT6**	46 (86.8)	4 (7.5)	2 (3.8)	0 (0.0)	1 (1.9)

[a]Fisher's exact p-value = 0.00050

Supplementary Table 7. Maternal and infant variables in association with breast milk microbiome cluster switching (n = 25).

**Maternal and Infant Variables**	***p*-value**
Maternal age[a]	0.50
Maternal pre-pregnancy BMI[a]	0.67
Gestational weight gain[a]	0.50
Parity > 1[b]	0.29
Prenatal antibiotics[b]	0.13
Peripartum antibiotics[b]	0.58
Postpartum antibiotics[b]	0.83
Infant sex[b]	0.39
Days postpartum of last sample collected	0.0093

[a]Variable modeled as continuous; *p*-value calculated with Kruskal-Wallis rank-sum test

[b]Variable modeled categorically, *p*-value calculated with Fisher's exact test

Supplementary Table 8. Maternal and infant variables in association with infant gut microbiome cluster switching (n = 27).

**Maternal and Infant Variables**	***p*-value**
Pre-pregnancy BMI[a]	0.39
Gestational weight gain[a]	0.80
Parity > 1[b]	0.42
Prenatal antibiotics[b]	0.22
Peripartum antibiotics[b]	0.88
Infant antibiotics at delivery[b]	1.00
Delivery mode[b]	1.00
Infant sex[b]	0.78
Infant age at last sample collection	0.11

[a]Variable modeled as continuous; *p*-value calculated with Kruskal-Wallis rank-sum test

[b]Variable modeled categorically, *p*-value calculated with Fisher's exact test

Supplementary Table 9. Paired infant gut microbiome cluster membership proportions by breast milk microbiome type.

All infants (n = 144)

	IGMT1	IGMT2	IGMT3	IGMT4
**BMT1**	4 (8.2)	11 (22.4)	22 (44.9)	12 (24.5)
**BMT2**	5 (13.9)	11 (30.6)	11 (30.6)	9 (25.0)
**BMT3**	10 (16.9)	19 (32.2)	27 (45.8)	3 (5.1)

Infants delivered by Cesarean Section (n = 40)

	IGMT1	IGMT2	IGMT3	IGMT4
**BMT1**	0 (0.0)	0 (0.0)	6 (54.5)	5 (45.5)
**BMT2**	1 (7.7)	0 (0.0)	6 (46.2)	6 (46.2)
**BMT3**	0 (0.0)	2 (12.5)	14 (87.5)	0 (0.0)

Infants delivered vaginally (n = 104)

	IGMT1	IGMT2	IGMT3	IGMT4
**BMT1**	4 (10.5)	11 (28.9)	16 (42.1)	7 (18.4)
**BMT2**	4 (17.4)	11 (47.8)	5 (21.7)	3 (13.0)
**BMT3**	10 (23.3)	17 (39.5)	13 (30.2)	3 (7.0)

Infants unexposed to formula (n = 108)

	IGMT1	IGMT2	IGMT3	IGMT4
**BMT1**	3 (8.1)	7 (18.9)	16 (43.2)	11 (29.7)
**BMT2**	4 (14.3)	10 (35.7)	8 (28.6)	6 (21.4)
**BMT3**	9 (20.9)	14 (32.6)	17 (39.5)	3 (7.0)

Supplementary Table 10. Association between most abundant breast milk taxon and most abundant infant stool taxon at 6-weeks post delivery (n = 144).

	G.Bacteroides	*G.Bifidobacterium*	*G.Clostridium sensu stricto*	*G.Escherichia/Shigella*	Other
G.Acinetobacter	5 (21.7)	6 (26.1)	1 (4.3)	3 (13.0)	8 (34.8)
G.Pseudomonas	11 (52.4)	8 (38.1)	1 (4.8)	1 (4.8)	0 (0.0)
G.Staphylococcus	4 (19.0)	5 (23.8)	1 (4.8)	5 (23.8)	6 (28.6)
G.Streptococcus	12 (27.9)	8 (18.6)	8 (18.6)	5 (11.6)	10 (23.3)
**Other**	9 (25.0)	7 (19.4)	1 (2.8)	7 (19.4)	12 (33.3)

Supplementary Table 11. Correlation between breast milk microbial community structure and infant gut microbial community structure in all mother-infant dyads.

Dyad timepoints	n	Crude Mantel Statistic	*p*-value	n	Adjusted[b] Mantel Statistic	*p*-value
6-week milk - 6-week infant stool	144	-0.032	0.88	113	-0.024	0.774
6-week milk - 4-month infant stool	15	0.021	0.41	10	-0.045	0.545
6-week milk - 6-month infant stool	14	0.303	0.015	12	0.528	0.001
6-week milk - 9-month infant stool	16	0.104	0.18	14	0.028	0.393
6-week milk - 12-month infant stool	108	0.003	0.47	84	0.014	0.372
4-month milk - 4-month infant stool	17	0.005	0.45	14	0.031	0.333
4-month milk - 6-month infant stool	16	0.191	0.058	15	0.209	0.041
4-month milk - 9-month infant stool	19	0.061	0.24	18	-0.01	0.503
4-month milk - 12-month infant stool	18	-0.045	0.59	16	0.14	0.162
6-month milk - 6-month infant stool	13	-0.033	0.58	11	-0.163	0.864
6-month milk - 9-month infant stool	15	0.257	0.052	13	0.337	0.023
6-month milk - 12-month infant stool	11	-0.017	0.50	11	-0.021	0.505
9-month milk - 9-month infant stool	14	0.12	0.13	13	0.189	0.068
9-month milk - 12-month infant stool	12	0.078	0.27	11	0.047	0.358
12-month milk - 12-month infant stool	5	-[a]	-[a]	4	-[a]	-[a]

[a]Dyad timepoint not tested due to n < 10

[b]Adjusted for maternal pre-pregnancy BMI, delivery mode, exposure to formula, exposure to antibiotics during pregnancy, maternal age, and infant sex

Supplementary Table 12. Correlation between breast milk microbial community structure and infant gut microbial community structure in mother-infant dyads where the infant is unexposed to formula.

		Crude			Adjusted[b]	
**Dyad timepoints**	**n**	**Mantel Statistic**	***p*-value**	**n**	**Mantel Statistic**	***p*-value**
6-week milk - 6-week infant stool	108	-0.029	0.82	96	-0.025	0.753
6-week milk - 4-month infant stool	13	-0.073	0.69	10	-0.045	0.548
6-week milk - 6-month infant stool	10	0.479	0.003	10	0.479	0.004
6-week milk - 9-month infant stool	11	0.173	0.12	11	0.166	0.138
6-week milk - 12-month infant stool	27	0.024	0.39	27	0.033	0.361
4-month milk - 4-month infant stool	14	-0.039	0.62	12	-0.009	0.47
4-month milk - 6-month infant stool	11	0.123	0.195	11	0.131	0.181
4-month milk - 9-month infant stool	12	-0.042	0.60	12	-0.044	0.611
4-month milk - 12-month infant stool	5	-[a]	-[a]	5	-[a]	-[a]
6-month milk - 6-month infant stool	10	-0.257	0.94	10	-0.271	0.955
6-month milk - 9-month infant stool	10	0.355	0.027	10	0.379	0.022
6-month milk - 12-month infant stool	4	-[a]	-[a]	4	-[a]	-[a]
9-month milk - 9-month infant stool	12	0.271	0.03	12	0.273	0.03
9-month milk - 12-month infant stool	4	-[a]	-[a]	4	-[a]	-[a]
12-month milk - 12-month infant stool	2	-[a]	-[a]	2	-[a]	-[a]

[a]Dyad timepoint not tested due to $n < 10$

[b]Adjusted for maternal pre-pregnancy BMI, delivery mode, exposure to antibiotics during pregnancy, maternal age, and infant sex

Supplementary Table 13. Correlation between breast milk microbial community structure and infant gut microbial community structure in mother-infant dyads delivered vaginally.

		Crude			Adjusted[b]	
**Dyad timepoints**	**n**	**Mantel Statistic**	***p*-value**	**n**	**Mantel Statistic**	***p*-value**
6-week milk - 6-week infant stool	104	-0.016	0.71	82	-0.009	0.59
6-week milk - 4-month infant stool	13	-0.018	0.52	8	-[a]	-[a]
6-week milk - 6-month infant stool	12	0.328	0.024	10	0.65	< 0.001
6-week milk - 9-month infant stool	14	-0.01	0.50	12	-0.231	0.958
6-week milk - 12-month infant stool	76	0.012	0.40	60	0.008	0.426
4-month milk - 4-month infant stool	15	0.031	0.37	12	0.062	0.29
4-month milk - 6-month infant stool	14	0.245	0.036	13	0.257	0.035
4-month milk - 9-month infant stool	17	0.08	0.24	16	-0.007	0.487
4-month milk - 12-month infant stool	16	0.00	0.446	14	0.235	0.105
6-month milk - 6-month infant stool	12	-0.038	0.59	10	-0.145	0.787
6-month milk - 9-month infant stool	14	0.273	0.059	12	0.417	0.014
6-month milk - 12-month infant stool	10	-0.098	0.612	10	-0.098	0.618
9-month milk - 9-month infant stool	12	0.027	0.39	11	0.1	0.244
9-month milk - 12-month infant stool	10	-0.044	0.575	9	-[a]	-[a]
12-month milk - 12-month infant stool	5	-[a]	-[a]	4	-[a]	-[a]

[a]Dyad timepoint not tested due to n < 10

[b]Adjusted for maternal pre-pregnancy BMI, exposure to formula, exposure to antibiotics during pregnancy, maternal age, and infant sex

Supplementary Table 14. Specific breast milk microbes are associated with infant gut microbes both cross-sectionally and longitudinally.

4-month milk vs. 9-month stool (n = 19 dyads)

Breast Milk Taxon	Infant Gut Taxon	rho	*p*-value	*q*-value
Granulicatella_2	*Clostridium_XVIII_1*	-0.81	2.4E-05	0.081
Granulicatella_1	*Dorea_2*	0.80	4.6E-05	0.081
Stenotrophomonas_2	*Bacteroides_11*	0.78	9.5E-05	0.086
Enterobacteriaceae_1	*Lachnospiraceae_19*	0.77	9.8E-05	0.086

4-month milk vs. 12-month stool (n = 18 dyads)

Breast Milk Taxon	Infant Gut Taxon	rho	*p*-value	*q*-value
Acinetobacter_5	*Erysipelotrichaceae_1*	-0.80	7.7E-05	0.095
Pseudomonas_5	*Streptococcus_5*	0.79	9.2E-05	0.095
Sphingobacterium_1	*Alistipes_2*	0.79	9.2E-05	0.095
Pantoea_1	*Clostridium_IV_4*	0.79	9.2E-05	0.095
Pantoea_1	*Lachnospiraceae_21*	0.79	1.1E-04	0.095

6-month milk vs. 6-month stool (n = 13 dyads)

Breast Milk Taxon	Infant Gut Taxon	rho	*p*-value	*q*-value
Streptococcus_1	*Intestinibacter_1*	0.90	2.8E-05	0.054
Parabacteroides_3	*Clostridium_sensu_stricto_5*	0.89	3.8E-05	0.054

6-month milk vs. 9-month stool (n = 15 dyads)

Breast Milk Taxon	Infant Gut Taxon	rho	*p*-value	*q*-value
Pantoea_1	*Lachnospiraceae_2*	0.99	2.5E-13	9.4E-10
Granulicatella_2	*Bacteroides_3*	0.87	2.1E-05	0.039
Pantoea_1	*Lachnospiraceae_9*	0.84	9.9E-05	0.094
Pantoea_1	*Lachnospiraceae_12*	0.84	9.9E-05	0.094

6-month milk vs. 12-month stool (n = 11 dyads)

Breast Milk Taxon	Infant Gut Taxon	rho	*p*-value	*q*-value
Clostridium_sensu_stricto_1	*Enterococcus_4*	0.99	1.4E-08	4.3E-05

9-month milk vs. 9-month stool (n = 14 dyads)

Breast Milk Taxon	Infant Gut Taxon	rho	*p*-value	*q*-value
Porphyromonas_3	*Streptococcus_5*	0.89	1.8E-05	0.043
Sphingomonas_4	*Veillonella_9*	0.88	3.8E-05	0.046
Sphingobium_2	*Blautia_5*	0.85	1.0E-04	0.081

Neisseria_4	*Veillonella_8*	0.84	1.8E-04	0.088
Methylobacterium_4	*Veillonella_9*	0.84	1.8E-04	0.088

1.2 Supplementary Figures

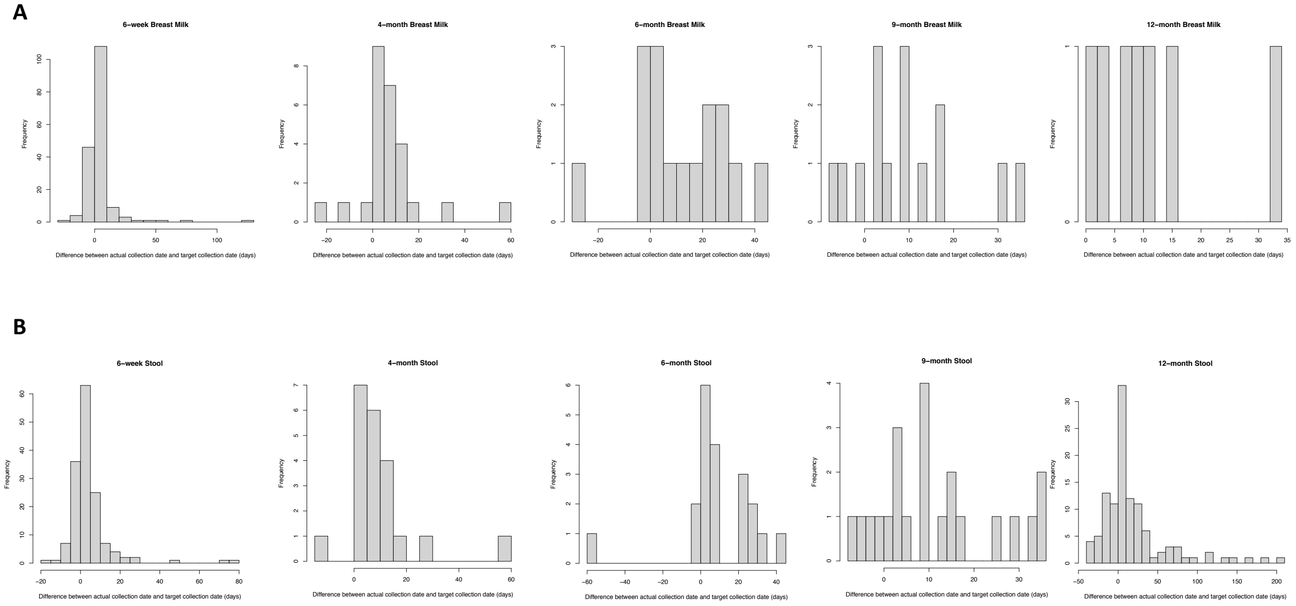

Supplementary Figure 1. Histograms showing the delta (days) between target sample collection time (exactly 6 weeks of age, etc.) and actual sample collection time for (A) breast milk and (B) infant stool.

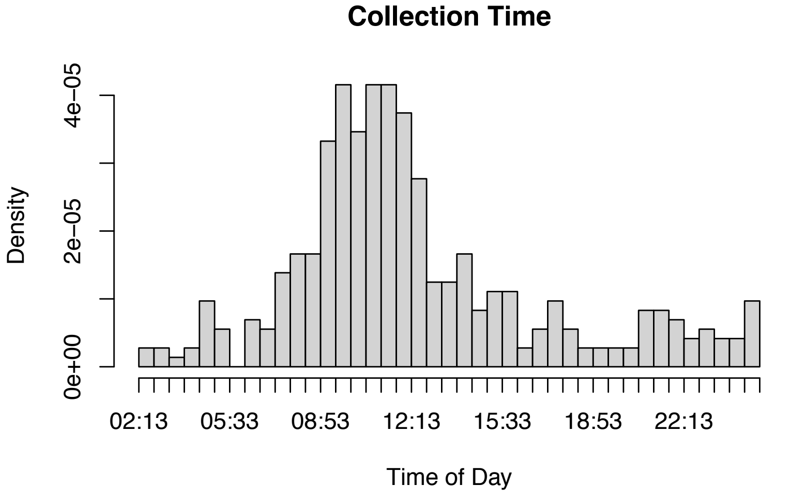

Supplementary Figure 2. Histogram showing the time of day of breast milk sample collection for a representative sample of women participating in the NHBCS.

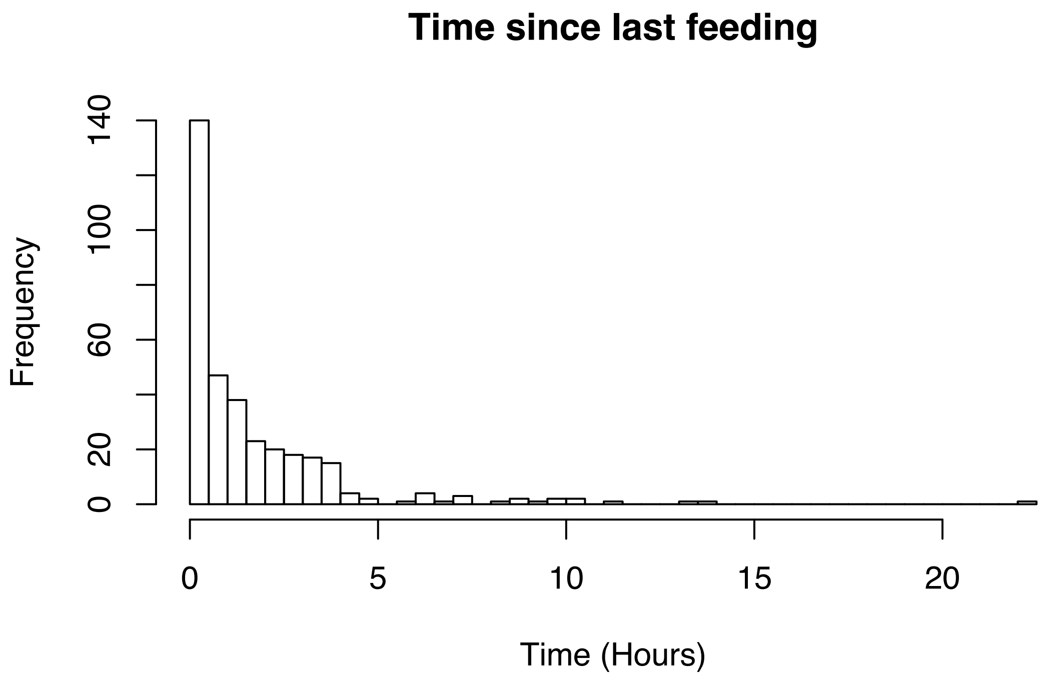

Supplementary Figure 3. The majority of milk samples were collected within a few hours of the last breastfeeding. Histogram showing the distribution of the time since last breastfeeding at the time of breast milk collection.

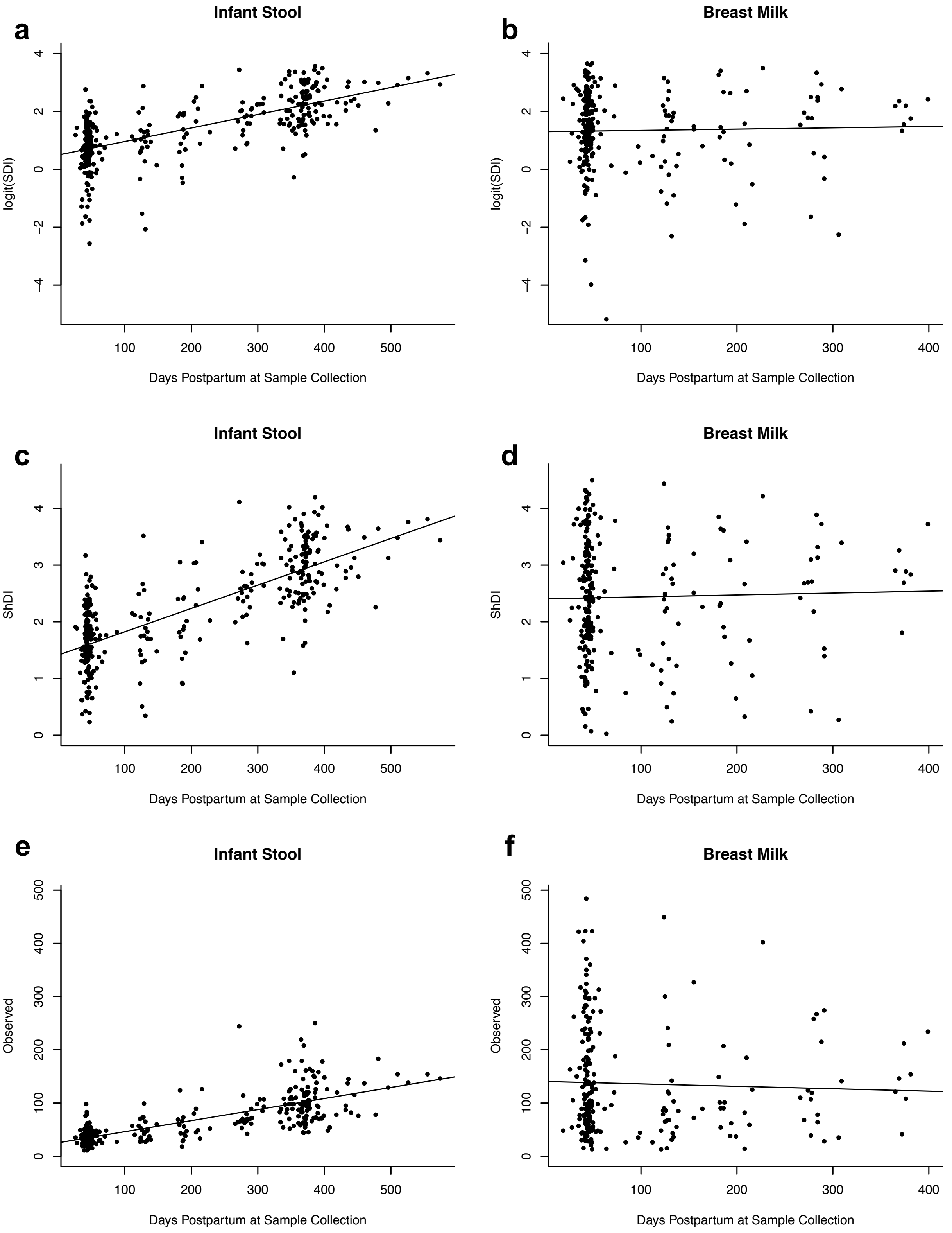

Supplementary Figure 4. Microbial alpha diversity over time based on continuous age at sample collection and non-rarefied data. Scatter plots showing Simpson's diversity index in (**A**) infant stool

and (**B**) breast milk, Shannon diversity index in (**C**) infant stool and (**D**) breast milk, and observed ASVs in (**E**) infant stool and (**F**) breast milk over the first year of life. Linear models with random effects for subject were fit modeling alpha diversity by age by sample collection.

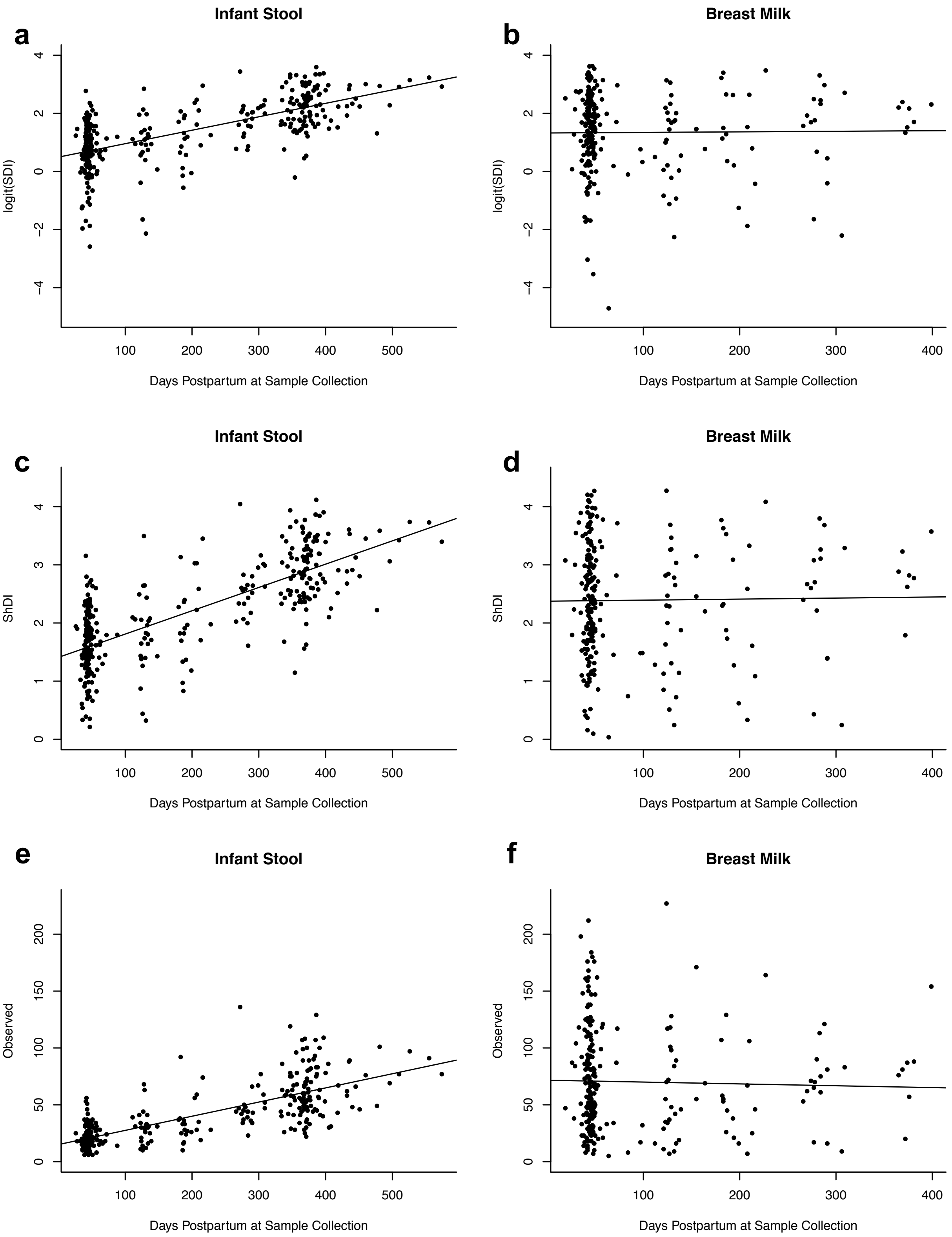

Supplementary Figure 5. Microbial alpha diversity over time based on continuous age at sample collection and rarefied data. Scatter plots showing Simpson's diversity index in (**A**) infant stool and

(**B**) breast milk, Shannon diversity index in (**C**) infant stool and (**D**) breast milk, and observed ASVs in (**E**) infant stool and (**F**) breast milk over the first year of life. Linear models with random effects for subject were fit modeling alpha diversity by age by sample collection.

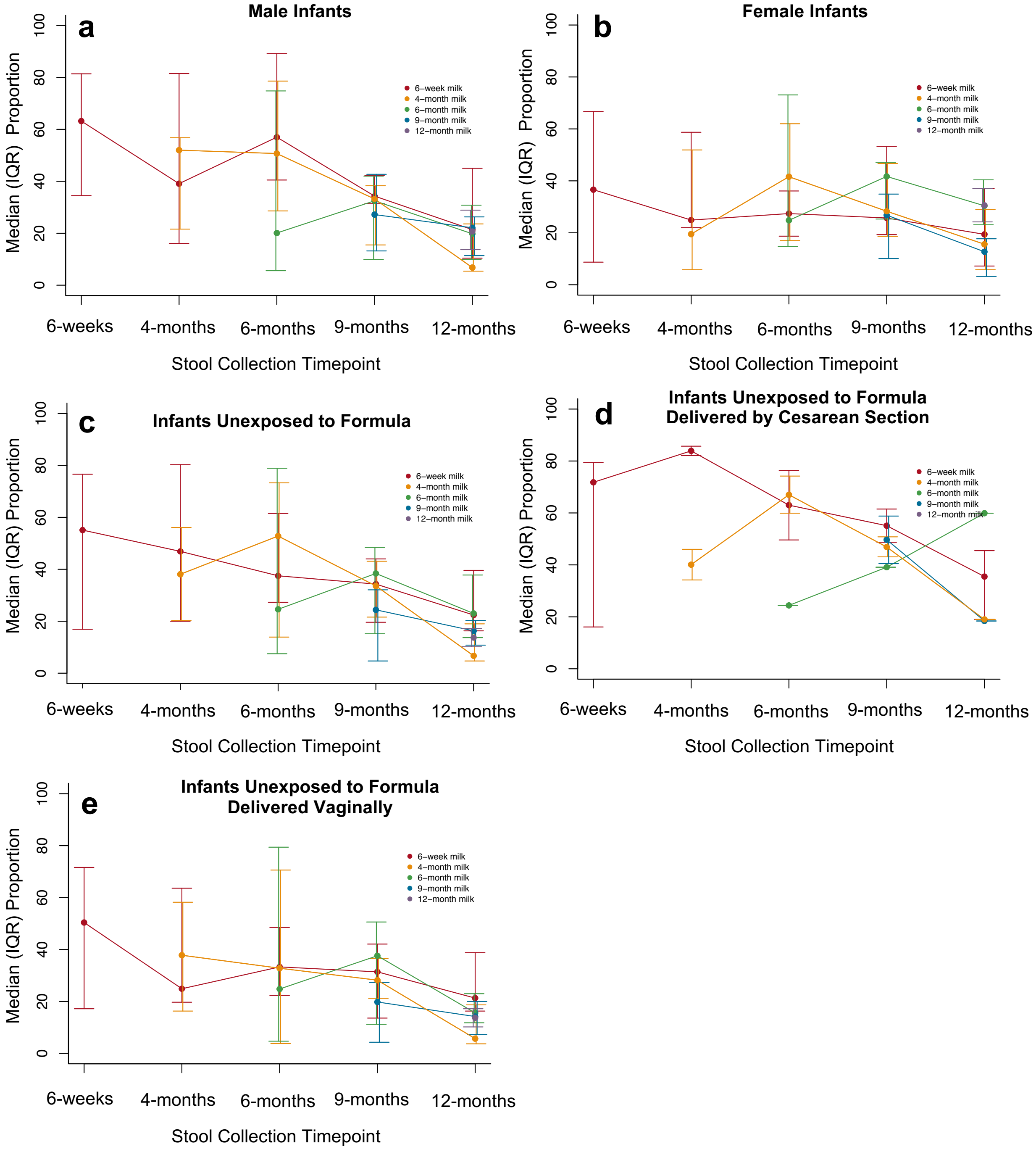

Supplementary Figure 6. Proportion of infant gut reads from ASVs in paired milk over time by infant sex, exposure to formula, and delivery mode. The median (IQR) of the total number of infant gut microbial reads from ASVs that also occurred in breast milk from an infant's mother divided by the total number of microbial reads in a sample for (**A**) male infants (Kolmogorov-Smirnov *p*-value = 0.0023 compared to random dyads), (**B**) female infants (Kolmogorov-Smirnov *p*-value = 0.95 compared to random dyads), (**C**) infants unexposed to formula (Kolmogorov-Smirnov *p*-value = 0.25 compared to random dyads), (**D**) infants unexposed to formula and delivered by Cesarean section

(Kolmogorov-Smirnov *p*-value = 0.042 compared to random dyads), and (**E**) infants unexposed to formula and delivered vaginally (Kolmogorov-Smirnov *p*-value = 0.35 compared to random dyads).

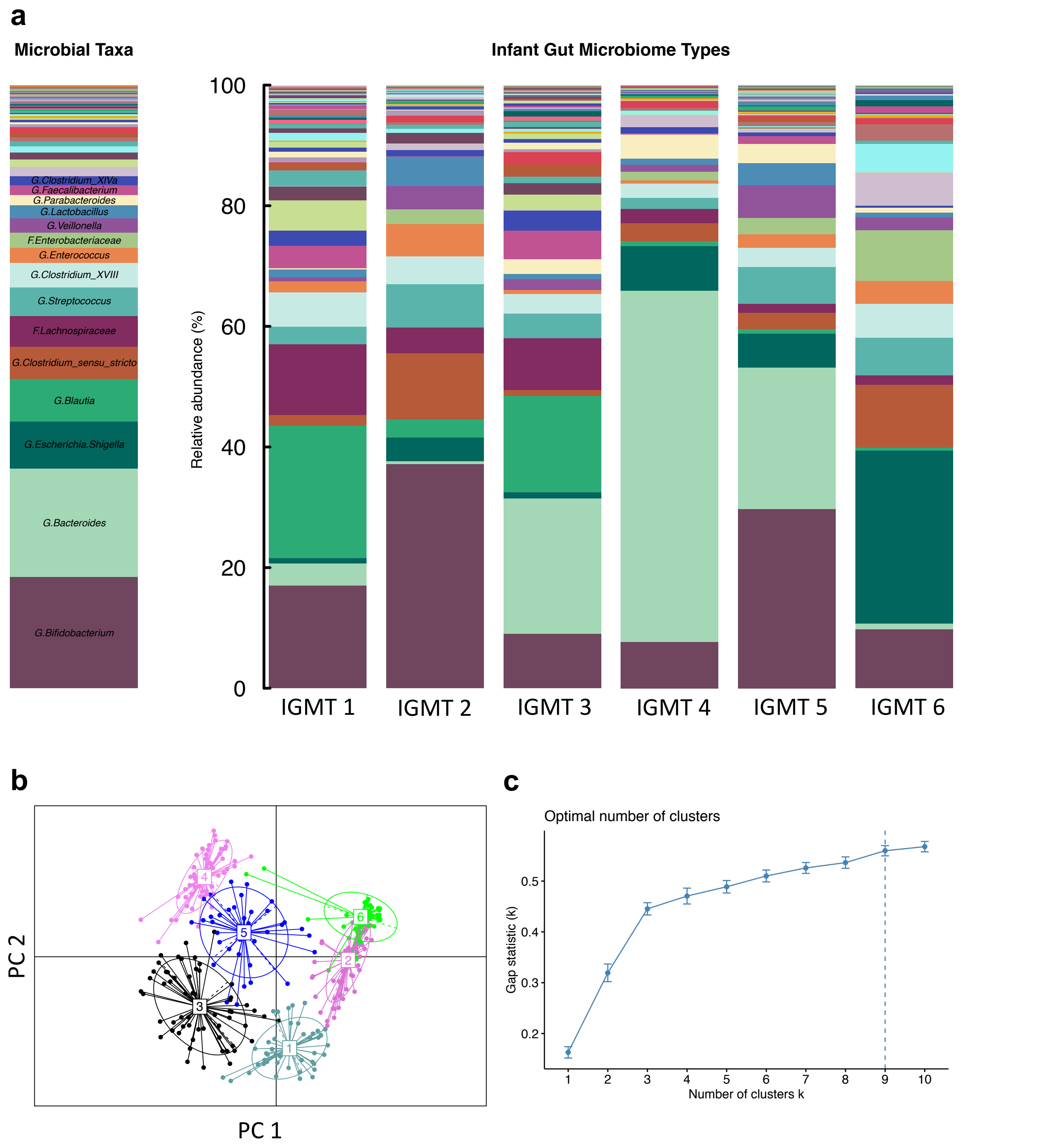

Supplementary Figure 7. Infant gut microbiome clusters including all timepoints through the first year of life. (**A**) Barplot of the average relative abundance of microbial taxa overall in all infant stool samples collected from approximately 6-weeks through one year of age, and by overall infant gut microbiome type (IGMT). (**B**) PCoA plot showing the separation of each cluster. (**C**) Plot showing the optimal number of clusters based on the gap statistic.

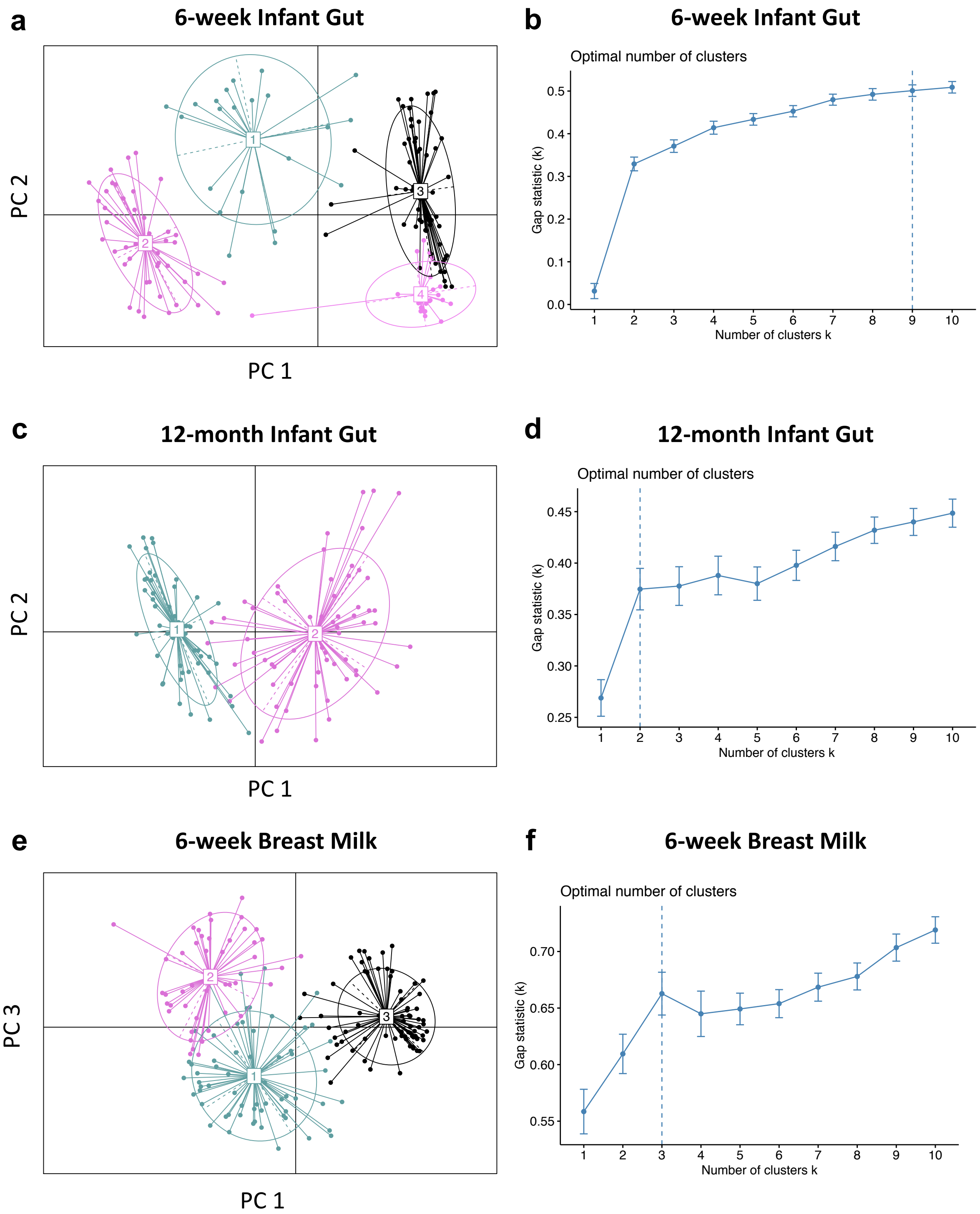

Supplementary Figure 8. Infant stool and breast milk cluster into distinct profiles at 6-weeks and 12-months. (**A**) PCoA with PC 1 vs. PC 2 for 6-week stool samples. (**B**) Plot showing the gap statistic for PAM clustering of 6-week infant gut samples. (**C**) PCoA with PC 1 vs. PC 2 for 12-month stool samples. (**D**) Plot showing the gap statistic for PAM clustering of 12-month stool samples. (**E**) PCoA with PC 1 vs. PC 3 for 6-week breast milk samples. (**F**) Plot showing the gap statistic for PAM clustering of 6-week breast milk samples.

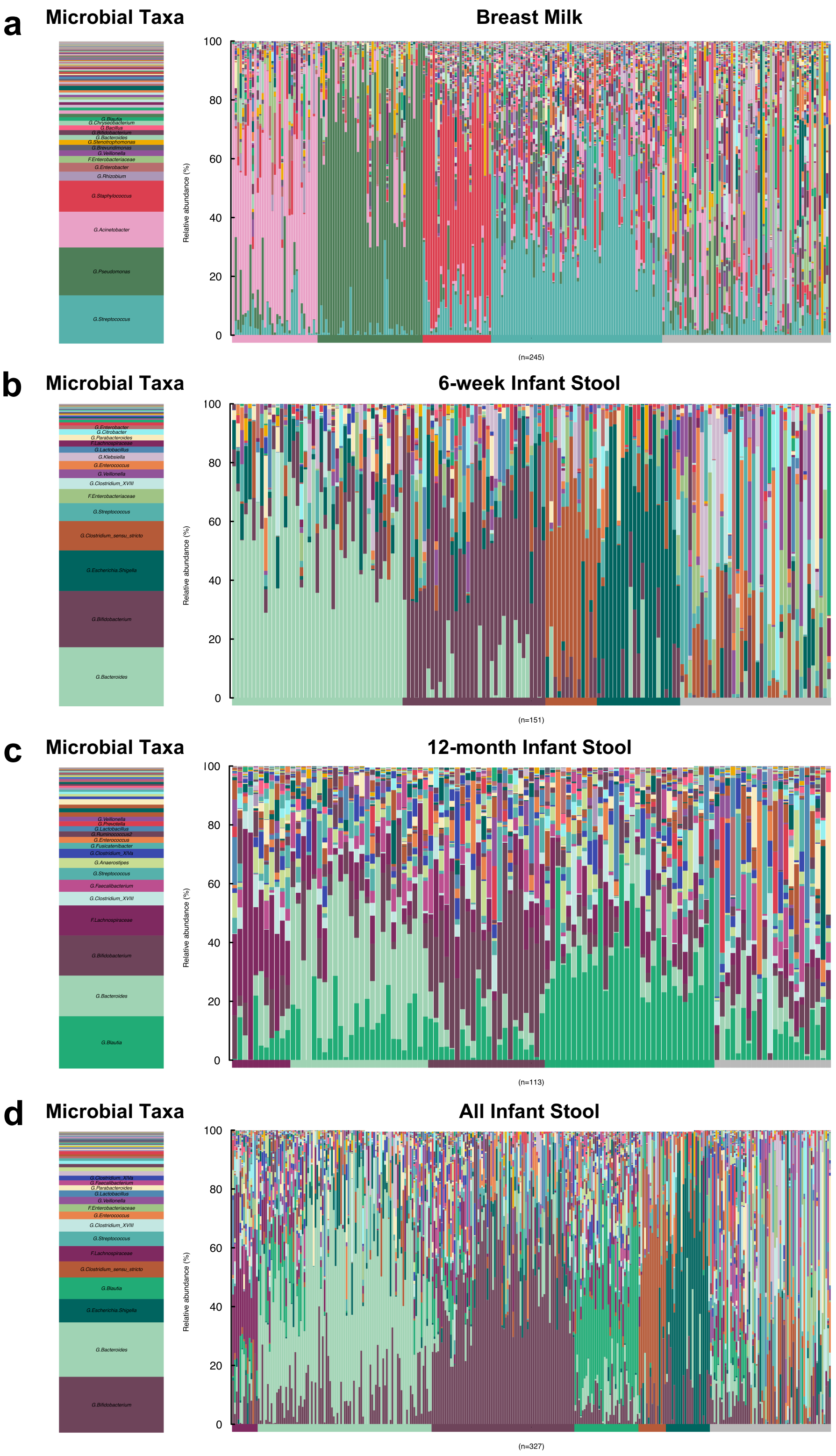

Supplementary Figure 9. Categorization of human milk and infant stool samples by the most abundant taxon. (**A**) Barplots showing the relative abundance of microbial taxa in milk samples

collected at all timepoints. The most abundant taxa were *Acinetobacter* (pink), *Pseudomonas* (green), *Staphylococcus* (red), *Streptococcus* (blue), or another taxon (grey). (**B**) Barplots showing the relative abundance of microbial taxa in infant stool samples collected at 6-weeks. The most abundant taxa were *Bacteroides* (mint), *Bifidobacterium* (purple), *Clostridium sensu stricto* (orange), *Escherichia/Shigella* (teal), or another taxon (grey). (**C**) Barplots showing the relative abundance of microbial taxa in infant stool samples collected at 12-months. The most abundant taxa were *Lachnospiraceae* (plum), *Bacteroides* (mint), *Bifidobacterium* (purple), *Blautia* (sea green), or another taxon (grey). (**D**) Barplots showing the relative abundance of microbial taxa in infant stool samples collected at all timepoints. The most abundant taxa were *Lachnospiraceae* (plum), *Bacteroides* (mint), *Bifidobacterium* (purple), *Blautia* (sea green), *Clostridium sensu stricto* (orange), *Escherichia/Shigella* (teal), or another taxon (grey).

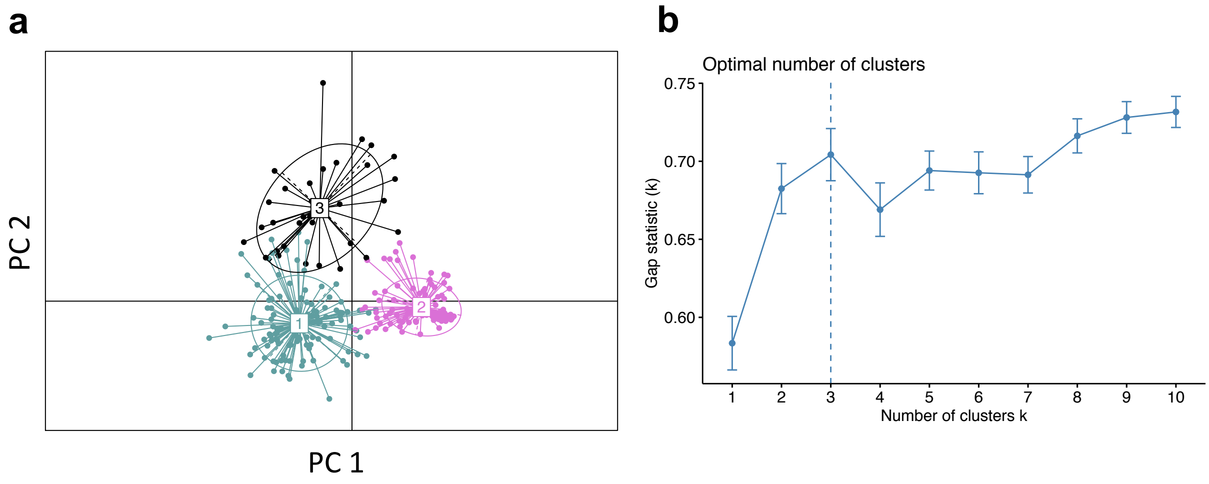

Supplementary Figure 10. Breast milk clusters into distinct profiles including all timepoints through the first year postpartum. (**A**) PCoA with PC 1 vs. PC 2 for all breast milk samples. (**B**) Plot showing the gap statistic for PAM clustering of all breast milk samples.

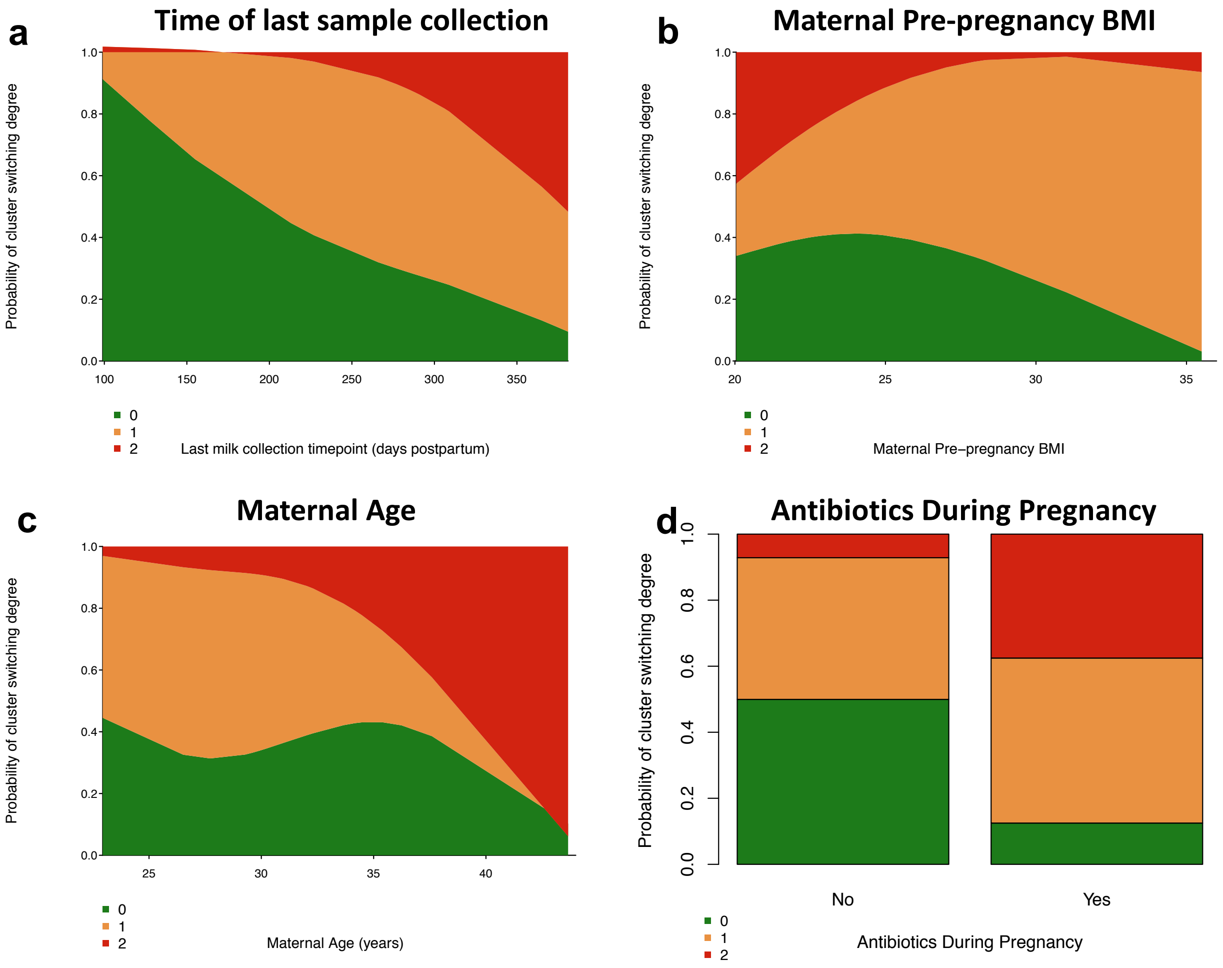

Supplementary Figure 11. Maternal factors and time postpartum of the last sample collection are associated with BMT cluster switching over the first year of life. Predicted probability plots for degree of breast milk cluster switching over the first of life by (**A**) the time of collection of the last breast milk sample, (**B**) maternal pre-pregnancy BMI, (**C**) maternal age, and (**D**) exposure to antibiotics during pregnancy.

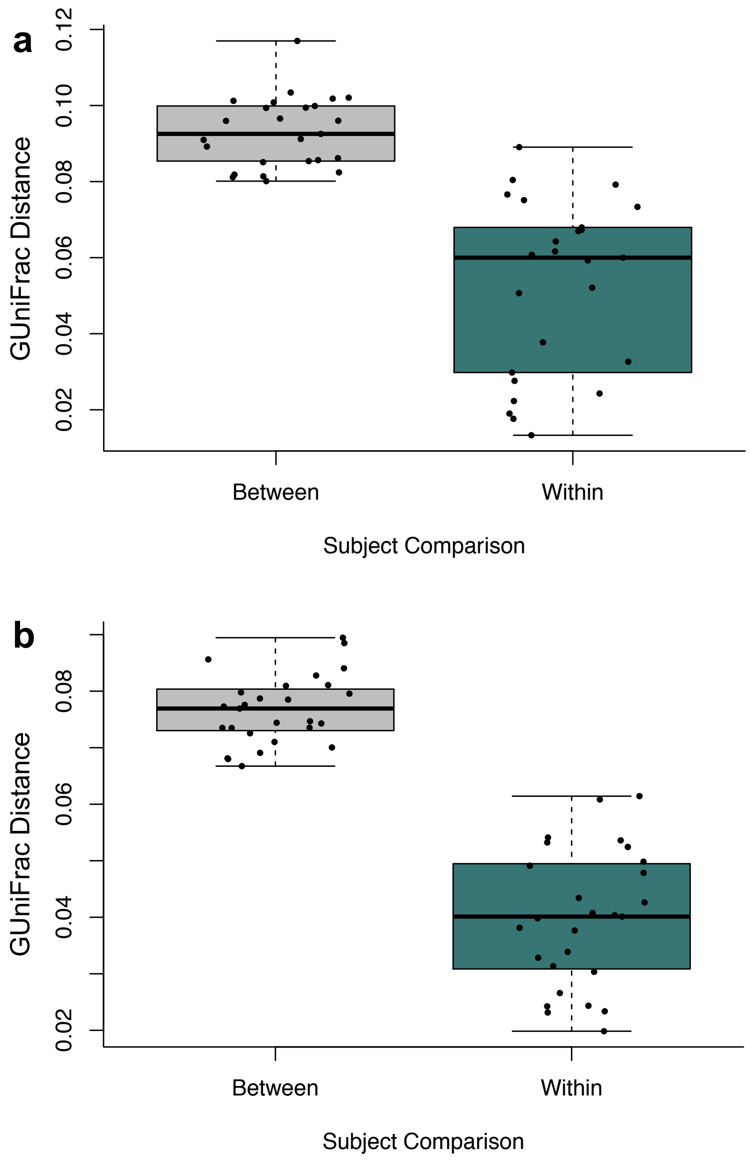

Supplementary Figure 12. GUniFrac distances are lower amongst samples from the same subject. Boxplots of the average GUniFrac distance within and between subjects with at least two longitudinally collected samples. (**A**) Average GUniFrac distances are lower between milk samples from the same woman compared to between subjects (Kruskal-Wallis rank-sum *p*-value = 4.38×10^{-9}). (**B**) Average GUniFrac distances are lower between stool samples from the same infant compared to between subjects (Kruskal-Wallis rank-sum *p*-value = 2.9×10^{-10}), excluding 12-month stool samples.

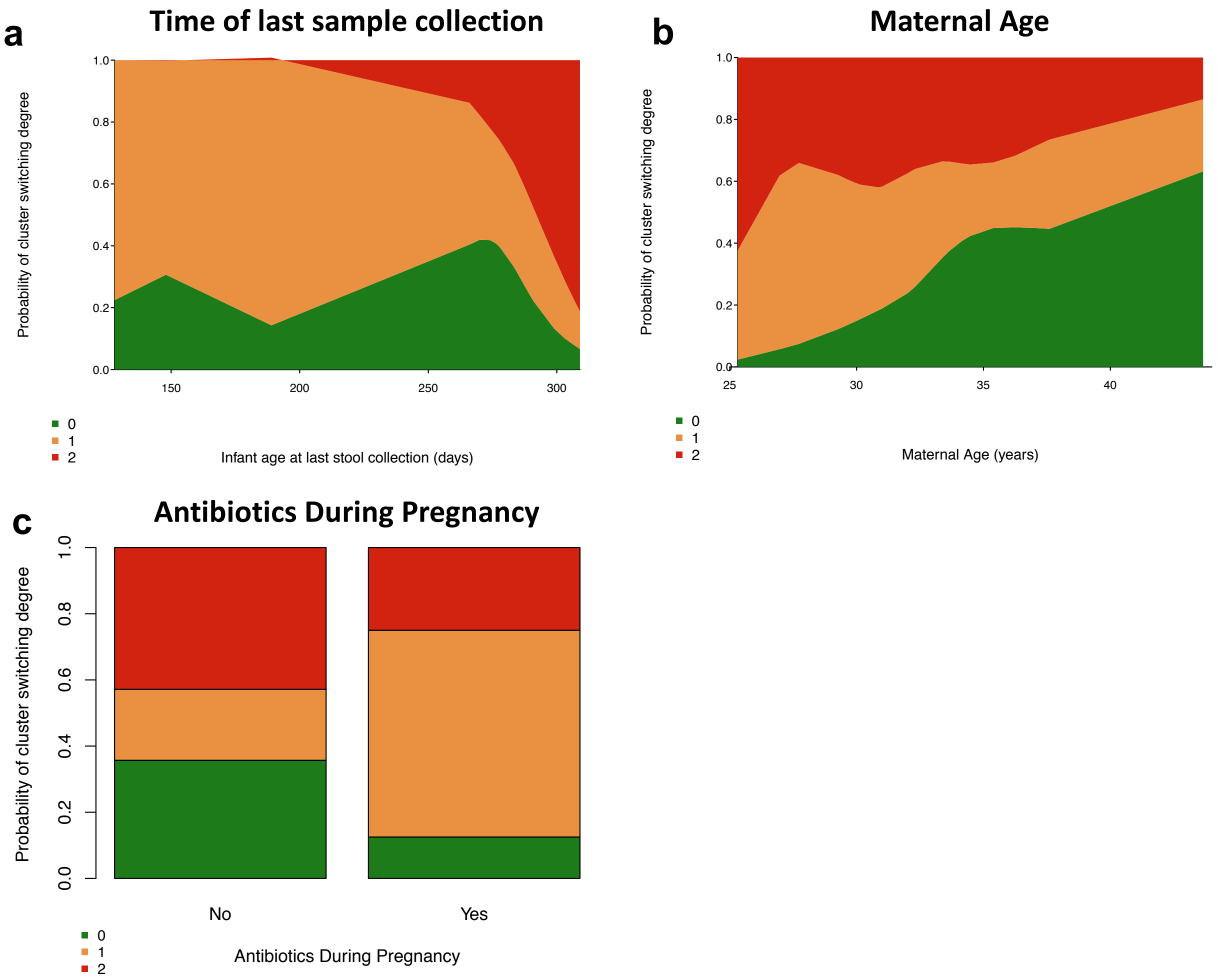

Supplementary Figure 13. Maternal factors and infant age at the last sample collection are associated with IGMT cluster switching over the first year of life. Predicted probability plots for degree of infant gut microbiome type cluster switching over the first of life by (**A**) the time of collection of the last infant stool sample, (**B**) maternal age, and (**C**) exposure to antibiotics during pregnancy.

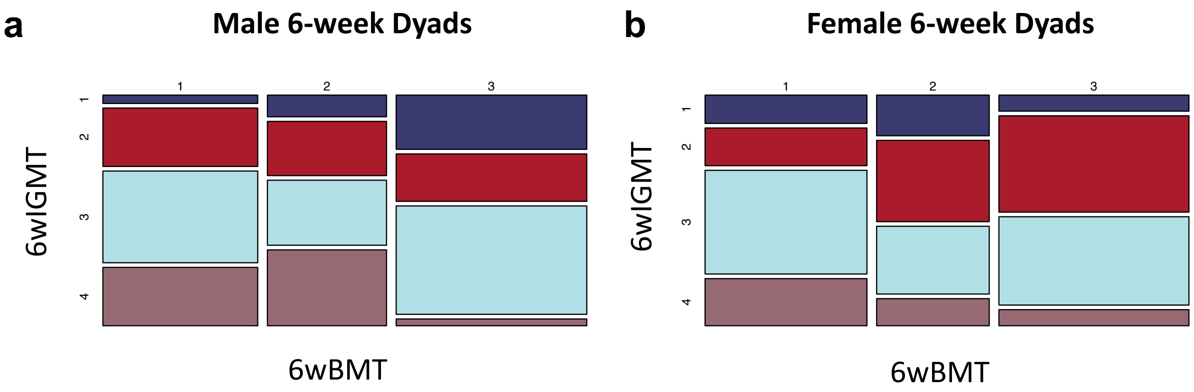

Supplementary Figure 14. Breast milk microbiome type is associated with infant gut microbiome type at 6 weeks of age only in male infants. **A**) Dyads with male infants (n = 78, Fisher's exact p-value = 0.020) and (**B**) dyads with female infants (n = 66, Fisher's exact p-value = 0.51).

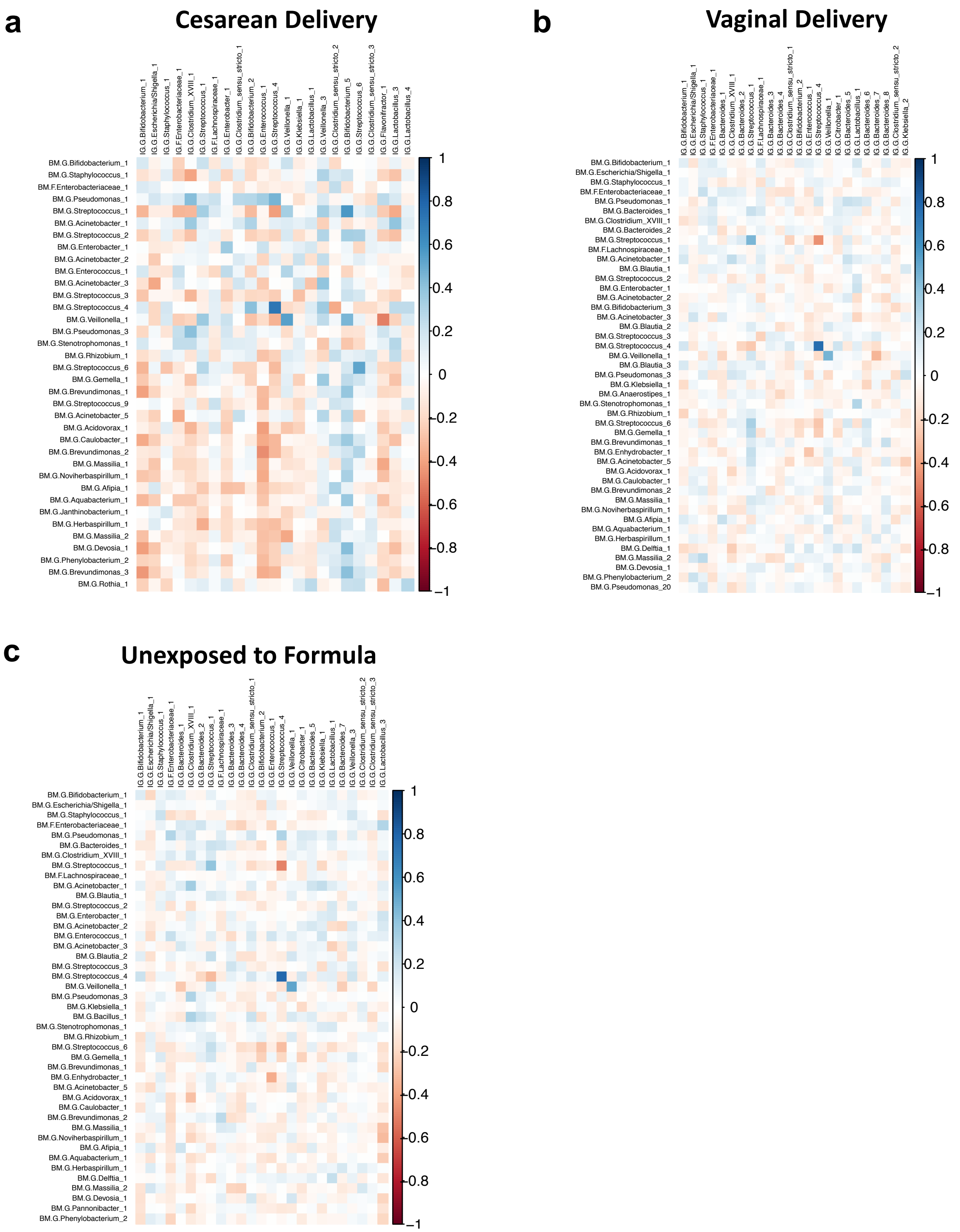

Supplementary Figure 15. Correlation between microbial taxa in contemporaneously collected 6-week breast milk and infant stool is stronger in the case of Cesarean delivery. (**A**) Spearman correlation between 6-week breast milk microbial taxa and 6-week infant gut microbial taxa in infants delivered by Cesarean section. (**B**) Spearman correlation between 6-week breast milk microbial taxa and 6-week infant gut microbial taxa in infants delivered vaginally. (**C**) Spearman

correlation between 6-week breast milk microbial taxa and 6-week infant gut microbial taxa in infants unexposed to formula.
